# A Safeguard Mechanism Regulates Rho GTPases to Coordinate Cytokinesis with the Establishment of Cell Polarity

**DOI:** 10.1371/journal.pbio.1001495

**Published:** 2013-02-26

**Authors:** Franz Meitinger, Heidi Richter, Sabrina Heisel, Birgit Hub, Wolfgang Seufert, Gislene Pereira

**Affiliations:** 1 Molecular Biology of Centrosomes and Cilia Unit, DKFZ-ZMBH Alliance, German Cancer Research Center, Heidelberg, Germany; 2 Department of Genetics, University of Regensburg, Regensburg, Germany; 3 Department of Tumor Virology, German Cancer Research Center, Heidelberg, Germany; Yale University, United States of America

## Abstract

The spatiotemporal control of cell polarity is crucial for the development of multicellular organisms and for reliable polarity switches during cell cycle progression in unicellular systems. A tight control of cell polarity is especially important in haploid budding yeast, where the new polarity site (bud site) is established next to the cell division site after cell separation. How cells coordinate the temporal establishment of two adjacent polarity sites remains elusive. Here, we report that the bud neck associated protein Gps1 (GTPase-mediated polarity switch 1) establishes a novel polarity cue that concomitantly sustains Rho1-dependent polarization and inhibits premature Cdc42 activation at the site of cytokinesis. Failure of Gps1 regulation leads to daughter cell death due to rebudding inside the old bud site. Our findings provide unexpected insights into the temporal control of cytokinesis and describe the importance of a Gps1-dependent mechanism for highly accurate polarity switching between two closely connected locations.

## Abstract 2

Gps1 provides a novel molecular polarity cue at the cell division site that guides Rho1- and Cdc42-dependent polarization during and after cytokinesis in budding yeast.

## Author Summary

In budding yeast, cell polarization (or the asymmetric distribution of subcellular components) ensures the targeted transport of proteins and membrane material to the sites of cell growth or cell division in late mitosis. Two conserved members of the Rho-GTPase family, Rho1 and Cdc42, are master regulators of cell polarity. While Rho1 has a well-established role in cytokinesis and cell separation, Cdc42 helps to establish the new polarity site from which the future daughter cell will grow after cytokinesis. Interestingly, despite the fact that Cdc42 is recruited to the site of cell division at the same time as Rho1, the new daughter cell never emerges from the site previously used for cytokinesis during the preceding cell cycle, and it remains elusive how cells coordinate the distinct functions of Rho1 and Cdc42 during cytokinesis. Here, we show that the novel protein Gps1 marks the cell division site, where it maintains Rho1-dependent polarity until cell separation is completed. We also demonstrate that Gps1 prevents activation of Cdc42 at the site of cell division during cytokinesis. We propose that Gps1 provides a novel polarity cue that guides the establishment of a new polarity site, away from the old site of cell division, where the new daughter cell then emerges.

## Introduction

Cell polarization, i.e., the asymmetric distribution of subcellular structures and components, is critical for a variety of biological processes in uni- and multicellular organisms [1]–[3]. Rho GTPases are the major evolutionarily conserved regulators of polarity in yeast and mammalian cells. Of these G-proteins, RhoA, Cdc42, and Rac1, in particular, play essential roles in establishing polarity in different contexts, including asymmetric cell division, wound healing, apical–basal polarity of epithelial cells, and front–rear polarity of migrating cells [3]–[5].

The activation of Rho GTPases is under tight spatiotemporal control in response to extra- or intracellular polarity cues (e.g., chemical gradients, cell–cell interactions, or other landmarks) during the establishment and maintenance of cell polarity [4]. In this context, membrane-associated proteins often mediate activation or inhibition of Rho GTPases [1],[2],[4],[6]. Another feature of polarity establishment and maintenance is that Rho GTPases can be either simultaneously activated at various locations to fulfill different functions (e.g., during cell migration) or sequentially activated at the same location through cross-talk mechanisms (e.g., during single cell wound healing) [5],[6]. Both mechanisms require elaborate systems that enable both temporal and spatial separation of Rho GTPase activation.

In budding yeast, the Rho GTPases Rho1 (RhoA homolog) and Cdc42 are essential for bud growth, mating, and cell separation. Although Rho1 and Cdc42 share common effectors (formins, exocyst), they play distinct roles during the establishment and maintenance of cell polarity [3]. Unlike Cdc42, Rho1 has a well-established role in cytokinesis and cell separation. During anaphase, the Rho1 guanine nucleotide exchange factor (GEF) Tus1 recruits Rho1 to the cell division site (bud neck), thereby promoting the formation of the contractile actomyosin ring (AMR), which drives membrane ingression and primary septum formation during cytokinesis [7],[8]. Rho1 remains at the cell division site after the AMR has contracted and is important for the final step of cell abscission [9]. In budding yeast, abscission is a two-step process that involves the formation of the secondary septum (cell wall deposition) and septum cleavage mediated by a set of hydrolases. Both processes rely upon targeted vesicle transport, and both are mediated by the Rho1 effectors Bni1 (formin) [10] and Sec3 (part of the exocyst complex) [11]. Cell wall deposition also depends on the Rho1 effector Fks1 (β-1,6-glucan-synthase) [12].

Although Cdc42 co-localizes with Rho1 at the bud neck during cytokinesis and abscission, its contribution to the physical process of cell separation remains elusive. After completion of cytokinesis, Cdc42 establishes the new polarity site, from which the future daughter cell (bud) will emerge, thereby determining the new cell polarity axis [3]. Interestingly, the new bud never emerges from the site used for cytokinesis during the preceding cell cycle, despite the fact that Cdc42 is recruited to the bud neck at the same time as Rho1. These observations raise important questions regarding the role that Cdc42 plays at the cell division site and whether mechanisms are in place to inhibit Cdc42-dependent bud emergence at the bud neck during cytokinesis. One possibility could be that Cdc42 is restrained in an inactive state at the cell division site. Alternatively, Cdc42 may activate effectors at the bud neck different from those employed at the new bud site. The scaffold protein Bem1 and the GEF Cdc24 are major regulators of Cdc42 localization and activation [3],[13]–[16]. Given that both molecules co-localize with Cdc42 at the bud neck and bud site [3], it is unlikely that they are responsible for the specificity of Cdc42 towards different effectors. Nevertheless, the shift of the Cdc42-associated polarity site suggests that regulatory mechanisms are in place to couple the establishment of a new cell polarity site with the successful completion of cytokinesis and cell separation. However, such mechanisms have not been reported so far.

Here, we show that the uncharacterized gene product of *YPL158C*, which we named Gps1 (GTPase-mediated polarity switch 1), functions as a novel polarity cue at the cell division site and is required for both Cdc42 and Rho1 function at this site. Our data show that Gps1 regulates both Rho1- and Cdc42-dependent signaling in parallel. Gps1 marks the closed cell division site post–AMR contraction and is required to maintain the Rho1-dependent polarization that is necessary for secondary septum formation and cell separation. At the same time, Gps1 specifically inhibits Cdc42-dependent activation of the p21-activated kinase Cla4 at the bud neck. This inhibition of Cla4 is essential to prevent budding inside the established cell division site. Failure in Gps1 regulation leads to the death of the daughter cell. We therefore propose that Gps1 is a critical polarity cue that confers a high degree of accuracy in polarity switching between two closely connected locations.

## Results

### Gps1 Interacts with Cdc42 and Rho1 and Forms a Disc-Like Structure at the Bud Neck

**Figure 1:**
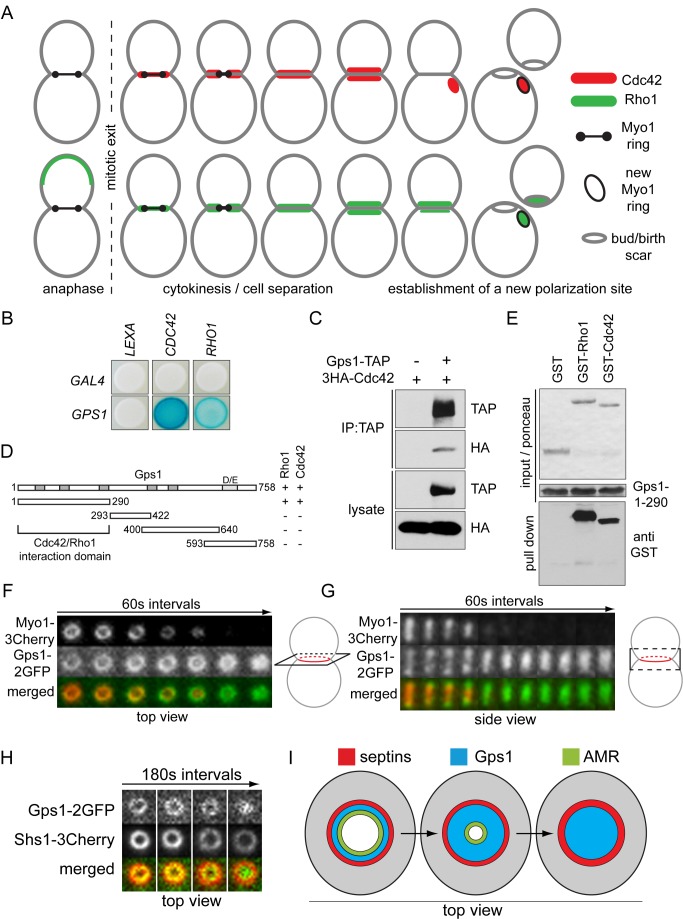
Gps1 interacts with Cdc42 and Rho1 and localizes at the cell division site. (A) Schematic representation of Cdc42 and Rho1 localization during and after cytokinesis. (B) Yeast two-hybrid interaction of Gps1 with Cdc42 and Rho1. (C) Co-immunoprecipitation (IP) between Cdc42 and Gps1. (D) Yeast two-hybrid interactions of Gps1 fragments with Cdc42 and Rho1. Grey boxes indicate conserved regions among Gps1 homologs. D/E, glutamate- and aspartate-rich region. Numbers indicate amino acid positions. (E) In vitro binding between Gps1-1-290 and Rho1 and Cdc42. (F and G) Still images of time-lapse microscopy showing co-localization of Myo1 and Gps1 during AMR contraction. Top (F) and side (G) views of the bud neck region are shown. (H) Still images of time-lapse microscopy showing co-localization of Gps1 with the septin Shs1. Only the magnified bud neck region (top view) is shown. (I) Overview on Gps1 localization during cytokinesis in comparison to AMR (Myo1) and septin complex (Shs1).

The highly conserved Cdc42 GTPase has a well-known function in polarity establishment during bud growth, which is consistent with its localization at the incipient bud site. However, Cdc42 forms a ring-like structure at the bud neck before AMR contraction, is reorganized as a disc-like structure after AMR constriction, and then splits into two well-separated signals just before cell separation (Figures 1 and S1A). This complex pattern of localization at the bud neck during cytokinesis implies that Cdc42 may either perform an important function during cytokinesis or be inhibited during AMR contraction. To understand the regulation and function of Cdc42, we screened for bud neck proteins that interact with Cdc42 using the yeast two-hybrid system. We identified the protein Gps1 as a putative Cdc42 interactor (Figure 1B). Gps1 is a protein of uncharacterized function that was previously reported to localize at the bud neck [17].

Co-immunoprecipitation using a pair-wise combination of functional tagged proteins confirmed that Gps1 and Cdc42 interacted with one another (Figure 1C). Interestingly, we observed that Gps1 also associated with the Rho GTPase Rho1 in the yeast two-hybrid system (Figure 1B). In addition, tryptic peptides corresponding to Rho1 were identified by mass spectrometry analysis of purified Gps1-TAP complexes (Figure S1C). To map the Cdc42 and Rho1 interaction domain of Gps1, we generated several Gps1 truncations (Figure 1D). We found that the N-terminal 290 residues of Gps1 (Gps1-1-290) were sufficient to interact with both Rho1 and Cdc42 in the yeast two-hybrid system (Figure 1D; data not shown). These interactions were most likely direct, as bacterially purified 6His-Gps1-1-290 associated with purified GST-Cdc42 or GST-Rho1 but not GST in vitro (Figure 1E). Interestingly, Gps1-1-290 bound to both GDP- and GTP-locked mutants of Cdc42 and Rho1 in the yeast two-hybrid system (Figure S2A and S2B) and in vitro (Figure S2C), indicating that Gps1 interacts with Cdc42 and Rho1 independently of their nucleotide-bound forms. Given that the temporal recruitment of Rho1 to the bud neck was similar to that of Cdc42 (Figures 1A, S1A, and S1B S1B), we postulated that both Rho1 and Cdc42 are functionally linked to Gps1.

Live cell imaging showed that Gps1 formed a ring-like structure at the bud neck that co-localized with Myo1 before the onset of AMR contraction (Figure 1F and 1G). However, Gps1 did not contract with the AMR. Instead, the Gps1 signal spread over the area defined by the initial ring and now formed a disc-like structure after AMR constriction. This area was confined by the septin ring (Figures 1H and S1D). The septin complex may therefore act as a diffusion barrier to maintain Gps1 at the bud neck region after AMR contraction, as previously suggested for other components [18]. Importantly, despite its localization with Cdc42 and Rho1 at the bud neck during cytokinesis, Gps1 differed from either partner protein, by not relocating to the new polarization site (Figure S1D), suggesting that Gps1 does not play any role at the new bud site. Together, the data indicate that Gps1 is a novel Cdc42- and Rho1-interacting protein that defines a unique micro-domain at the bud neck region that might have a specific role in late steps of cytokinesis and cell separation (Figure 1I).

### Gps1 Has a Role in Cytokinesis

**Figure 2:**
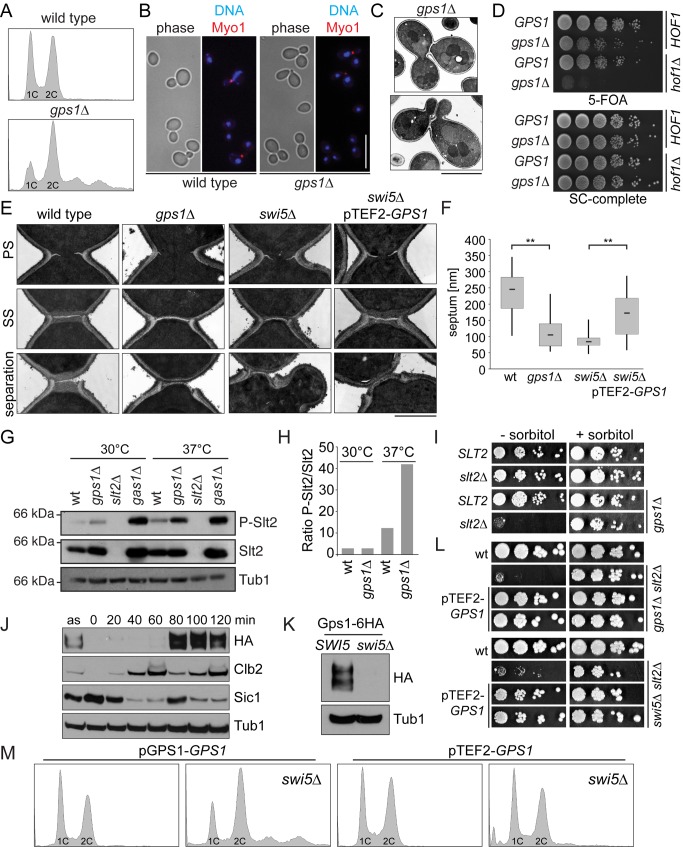
Gps1 is required for cytokinesis. (A) The FACS profiles show the DNA content distribution of wild-type and *gps1Δ* cells. (B) Still images showing the multiple bud phenotype (phase contrast), myosin II (Myo1-GFP), and DNA (DAPI) of wild-type and *gps1Δ* cells. Scale bar: 10 µm. (C) Electron micrographs of *gps1Δ* cells. Scale bar: 4 µm. (D) Serial dilutions of the indicated genotypes carrying *HOF1* on an *URA3*-based plasmid were spotted on the indicated plates. 5-FOA selects against the *URA3* plasmid. Lack of growth on 5-FOA indicates synthetic lethality. (E) Electron micrographs showing different cytokinesis steps of the indicated genotypes (note that only the bud neck regions are shown). PS, primary septum; SS, secondary septum. Scale bar: 1 µm. (F) Quantification of septum thickness (primary septum+secondary septum) of wild-type (wt; *n* = 33), *gps1Δ* (*n* = 47), *swi5Δ* (*n* = 28), and *swi5Δ* pTEF2-*GPS1* cells. “**” indicates *p*<0.0001. (G) Immunoblot showing phosphorylated (P-Slt2) and total Slt2 levels of the indicated strains. Tub1 served as a loading control. (H) Quantification of (G). (I) Genetic interaction between *GPS1* and *SLT2* tested as in (D), but using plates lacking or containing sorbitol for osmo-stabilization. (J) Time-course experiment from G1 arrested cells (alpha-factor, *t* = 0) showing steady-state levels of Gps1-6HA. Clb2 and Sic1 were used as markers for mitotic exit. Tub1 served as a loading control. as, asynchronous culture. (K) Gps1-6HA protein levels in wild-type and *swi5Δ* cells. (L) The genetic lethality between *slt2Δ* and *gps1Δ* (top panel) and between *slt2Δ* and *swi5Δ* (bottom panel) can be rescued by expressing *GPS1* from the Swi5-independent TEF2 promoter (pTEF2-*GPS1*). (M) FACS profiles of the indicated cell types.

To investigate whether Gps1 plays a role in cytokinesis, we analyzed the phenotype of *GPS1* knock-out (*gps1Δ*) cells. The absence of *GPS1* gave rise to cells carrying more than the regular 2C amount of DNA, and a large proportion of mother cells retained a connection to two or more buds (Figure 2A–2C). This phenotype was rarely seen in wild-type cells (frequency of less than 0.2%, in comparison to 40% in *gps1Δ* cells) and strongly indicates that cell separation was delayed in the absence of *GPS1*. We also found that *gps1Δ* cells were unable to survive in the absence of the cytokinetic component *HOF1* (*gps1Δ hof1Δ*; Figure 2D, lanes 3 and 4). Hof1 plays an important role in coordinating AMR contraction with septum formation and cytoskeleton polarization toward the cell division site during cytokinesis [19],[20]. The synthetically lethal interaction between *GPS1* and *HOF1* therefore indicates that Gps1 may work in a parallel pathway to Hof1 in promoting AMR contraction and/or septum formation during cytokinesis.

Through the inspection of AMR behavior and primary septum formation by live cell imaging and transmission electron microscopy (TEM), we excluded the possibility that Gps1 plays a role in primary septum biogenesis and/or AMR constriction (Figure S3). Importantly, TEM analysis revealed that formation of secondary septum cell wall material was strongly reduced in *gps1Δ* cells (Figure 2E and 2F). Despite this defect, *gps1Δ* cells eventually separated from each other, giving rise to newborn cells that had a 2- to 3-fold reduction in the thickness of the layer of secondary septal material that had been deposited at the bud/birth scar (the site at which cytokinesis took place) (Figure 2E and 2F). We hypothesized that the thinning of the secondary septum at the bud/birth scar in *gps1Δ* cells would engage the cell wall integrity (CWI) pathway. The CWI pathway is a mitogen-activated protein (MAP) kinase cascade that is activated upon cell wall stress to ensure cell survival by up-regulating genes that play important roles in cell wall synthesis and repair [21]. This assumption was confirmed by the observation that both protein levels and phosphorylated forms of the CWI-MAP kinase Slt2 were up-regulated in *gps1Δ* cells, as described for cells lacking *GAS1* (a 1,3-beta-glucanosyltransferase involved in the synthesis of the cell wall) [22] (Figure 2G and 2H). In addition, the survival of *gps1Δ* cells was dependent on *SLT2* (Figure 2I). Together, these data established that Gps1 is required for secondary septum assembly at the bud/birth scar.

### The Swi5 Transcriptional Factor Plays a Role in Cytokinesis through Gps1

The expression of *GPS1* has been previously shown to be under the control of the transcription factor Swi5 [23]. Swi5 is activated at the transition from mitosis into the G1 phase of the cell cycle [24]. Accordingly, Gps1-6HA protein levels strongly increased as cells exited mitosis, as monitored by the decline in the level of the mitotic cyclin Clb2 and the accumulation of the mitotic cyclin inhibitor Sic1, whose expression is also under Swi5 control [25] (Figure 2J). In addition, Gps1-6HA was depleted from cells lacking *SWI5* (Figure 2K), confirming that Swi5 controls *GPS1* expression in a cell-cycle-dependent manner.

The deletion of *SWI5* impeded secondary septum formation (Figure 2E and 2F), engaged the CWI pathway (Figure 2L), and led to the accumulation of cells with a DNA content that exceeded 2C (Figure 2M), in a manner that was comparable to *GPS1* deletion. We therefore postulated that *GPS1* might be the most important, if not the sole, gene under Swi5 control that is required for cytokinesis. In agreement with our hypothesis, the thickness of the secondary septum, the lethality of *swi5Δ slt2Δ* cells, and the accumulation of multi-nucleated cells were all rescued by expressing *GPS1* from a Swi5-independent promoter (pTEF2-*GPS1*; Figure 2E, 2F, 2L, and 2M). Taken together, these findings point to an important role of Swi5 in cytokinesis via regulation of *GPS1* expression that is manifested in a cell-cycle-dependent manner.

### Gps1 Controls Secondary Septum Formation through Rho1

**Figure 3:**
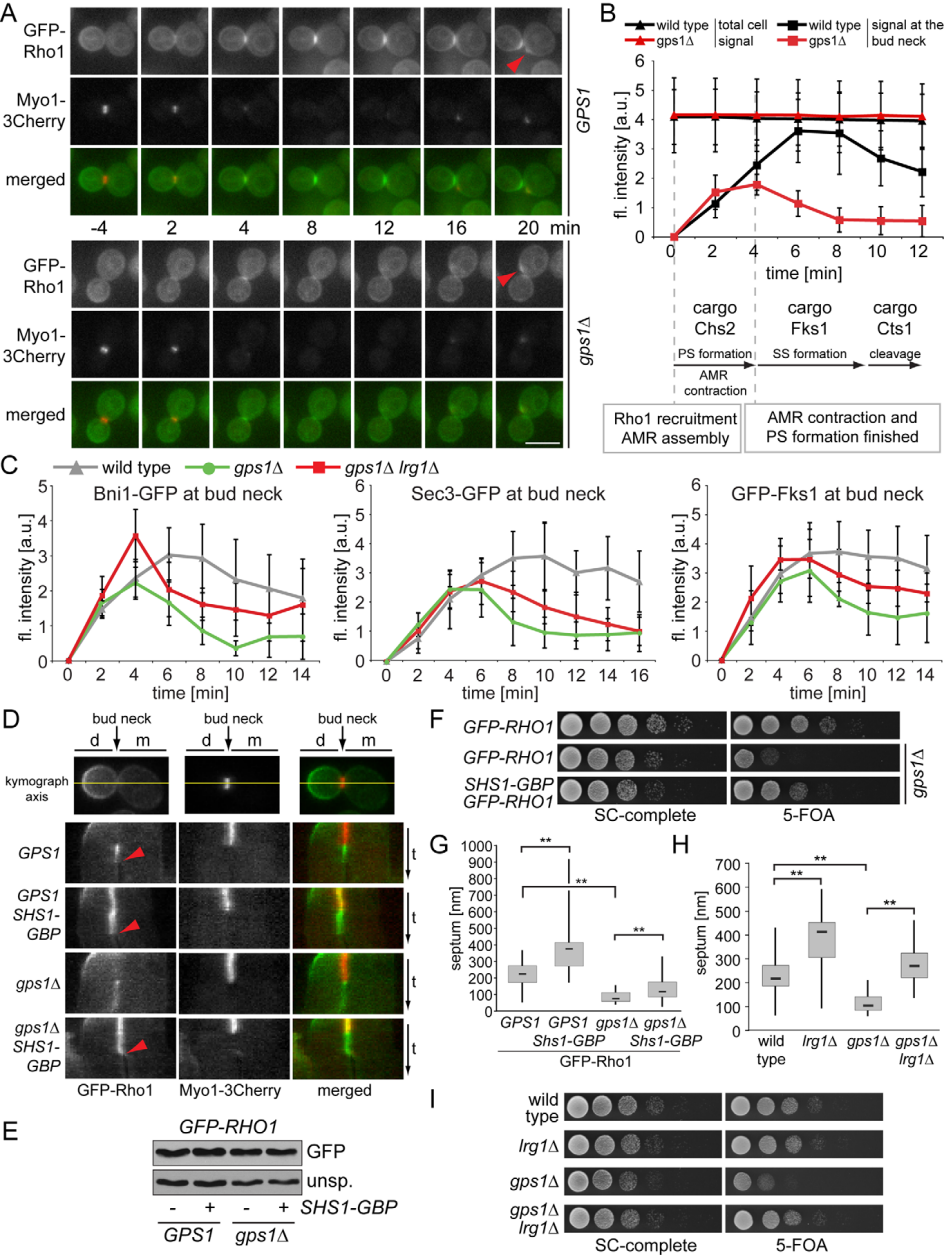
Gps1 regulates Rho1 at the cell division site. (A) Time-lapse series of GFP-Rho1 and Myo1-3Cherry at the bud neck in *gps1Δ* and wild-type cells. Red arrowhead indicates the new polarization site. (B) Quantification of (A) showing GFP-Rho1 fluorescence levels in wild-type (*n* = 6) and *gps1Δ* (*n* = 6) cells. Cytokinesis events are indicated. Chs2, Fks1, and Cts1 are cargos of the secretory pathway. (C) Quantification of the levels of bud-neck-associated Bni1-GFP (wild-type [*n* = 6], *gps1Δ* [*n* = 7], *gps1Δ lrg1Δ* [*n* = 8]), Sec3-GFP (wild-type [*n* = 5], *gps1Δ* [*n* = 5], *gps1Δ lrg1Δ* [*n* = 5]), and Fks1-GFP (wild-type [*n* = 5], *gps1Δ* [*n* = 4], *gps1Δ lrg1Δ* [*n* = 5]). (D) Continuous tethering of GFP-Rho1 to the bud neck with Shs1-GBP. Kymographs of the mother (m)–daughter (d) cell axis (yellow line) of the indicated genotypes are shown. The red arrowhead indicates the time of cell splitting, as determined by the loss of fluorescence signal between mother and daughter cell. (E) The immunoblot shows GFP-Rho1 levels in *GPS1*, *GPS1 SHS1-GBP*, *gps1Δ*, and *gps1Δ SHS1-GBP* cells. An unspecific (unsp.) signal was used as a loading control. (F) Serial dilutions of the indicated cell types carrying *GPS1* on an *URA3*-based plasmid. (G) Septum thickness of GFP-Rho1-containing *GPS1* (*n* = 34), *GPS1 SHS1-GBP* (*n* = 25), *gps1Δ* (*n* = 45), and *gps1Δ SHS1-GBP* (*n* = 46) cells. (H) Septum thickness of wild-type (*n* = 42), *gps1Δ* (*n* = 48), *lrg1Δ* (*n* = 26), and *gps1Δ lrg1Δ* (*n* = 32) cells. (I) Serial dilutions of the indicated cell types carrying *GPS1* on an *URA3*-based plasmid. Error bars in (B) and (C) show the standard deviation. “**” indicates *p*<0.0001. Scale bars: 5 µm. a.u., arbitrary units; fl. intensity, fluorescence intensity; PS, primary septum; SC, synthetic complete medium; SS, secondary septum.

The interaction between Gps1 and Rho1 suggested that Gps1 controls secondary septum formation via activation of Rho1. Rho1 activates the cell-wall- and septum-synthesizing Fks1 and two proteins that target this transmembrane protein to the bud neck: the formin Bni1 and the exocyst landmark protein Sec3. To determine whether Gps1 does indeed regulate Rho1, we monitored the distribution of Rho1. For this, we used a *GFP-RHO1* fusion expressed from the Rho1 endogenous promoter in a strain carrying untagged Rho1. Our GFP-Rho1 is partially functional, as it could promote the growth of Rho1-depleted cells once expressed at higher expression levels (Figure S4). As previously reported [9], GFP-Rho1 was recruited to the bud neck shortly before the onset of AMR contraction and persisted there until the cells separated (Figure 3A). This temporal bud neck association is consistent with the role of Rho1 in AMR assembly/contraction as well as in secondary septum formation [7],[12]. In *gps1Δ* cells, Rho1 was recruited to the bud neck shortly before AMR contraction, as in wild-type cells (Figure 3A and 3B; Video S1). Moreover, Rho1 accumulated to similar levels at the bud necks of both wild-type and *gps1Δ* cells (Figure 3A and 3B; Video S2), which is consistent with our observation that AMR and primary septum assembly were not affected by *GPS1* deletion (Figures 2E and S3). However, whereas Rho1 levels increased at the bud neck of wild-type cells after AMR contraction, they declined sharply in *gps1Δ* cells and only reappeared at the tip of the newly emerging bud (Figure 3A [arrowhead] and 3B; Videos S1 and S2). This reduced bud neck sequestration was also found for the Rho1 effectors Bni1, Sec3, and Fks1 (Figure 3C). Consequently, actin and myosin polarization toward the bud neck was, in general, impaired after AMR ring contraction in *gps1Δ* mutants (Figure S5A–S5F). These data suggest that *gps1Δ* cells cannot maintain Rho1 and downstream targets at the bud neck after the initial wave of polarization that controls Rho1 recruitment to promote AMR and primary septum functions.

We postulated that the levels of Rho1 at the bud neck after AMR contraction in *gps1Δ* cells would be insufficient to promote efficient Fks1 targeting and activity, thereby explaining the marked reduction of secondary septum material in these cells. In line with this assumption, *FKS1* overexpression partially rescued *gps1Δ* defects in secondary septum formation (Figure S5G). One prediction arising from this observation is that increasing Rho1 activity or tethering Rho1 to the bud neck independently of Gps1 function should rescue the secondary septum defect of *gps1Δ* cells. We observed that low overexpression of *RHO1* partially rescued the growth defect of *gps1Δ* cells (Figure S6A; Table S1 provides an overview of genetic manipulations that rescued *gps1Δ* defects), whereas higher levels of *RHO1* caused a toxic effect (Figure S6B). In addition, we found that the rapid cycling mutant Rho1-C25A (expressed from the Rho1 endogenous promoter), but not wild-type or GTP-locked Rho1, partially rescued the growth and secondary septum formation defects of the *gps1Δ* cells (Figure S6C–S6F) [9]. Next, we targeted Rho1 artificially to the bud neck in *gps1Δ* cells using the GFP-binding protein (GBP) strategy [26]. *GFP-RHO1* wild-type, GTP-locked, or rapid cycling mutants [9],[27] were expressed in cells in which the septin Shs1 was fused to GBP. This constitutively tethered Rho1 at the bud neck both in *SHS1-GBP* and *SHS1-GBP gps1Δ* cells (Figure 3D and 3E). Whereas GFP-Rho1 was mainly lost from the bud neck in *gps1Δ* cells after AMR contraction, GFP-Rho1 remained at the bud neck in *GPS1*, *GPS1 SHS1-GBP*, and *gps1Δ SHS1-GBP* cells until the cells had completed cell separation (Figure 3D, red arrowheads indicate the time point of cell separation). The growth defect, the reduction in secondary septum assembly, and the impaired Rho1 effector localization in *gps1Δ* cells were all partially rescued by bud neck targeting of GFP-Rho1 via Shs1-GBP (Figures 3F, 3G, S6G, and S6H). In this context, rapid cycling Rho1 mutants rescued the growth defect of *gps1Δ* mutants even better than wild-type Rho1, whereas GTP-locked mutants showed no or just a little rescue ability (Figure S6D–S6F). A similar inability of GTP-locked Rho1 mutants to rescue cytokinesis defects was reported previously [9]. Importantly, the increased bud neck targeting of the other bud-neck-residing Rho-GTPases, Rho2, Rho4, and Cdc42, failed to rescue the growth of *gps1Δ* cells (Figure S7A–S7D). This indicated that Rho2 and Rho4 did not influence the bud neck localization of Rho1 effectors, which is in line with the observation that the deletion of neither *RHO2* nor *RHO4* impaired the recruitment of Sec3 to the bud neck (Figure S7E). These findings highlight the specificity of Gps1 function in promoting Rho1 bud neck association.

Collectively, our data indicate that Gps1 is required to maintain Rho1 at the bud neck after AMR contraction, thereby contributing to the later functions performed by Rho1 in secondary septum formation and cell separation.

### Gps1 Regulates Rho1 Bud Neck Localization by Mechanisms That Are Independent of the Rho1 GEFs Tus1 and Rom2

We next assessed how Gps1 promotes the retention of Rho1 at the bud neck. The recruitment of Rho1 to the bud neck in late anaphase has been shown to depend upon GEF proteins, which themselves are targeted to the bud neck in a cell-cycle-dependent manner. During anaphase, the GEF Tus1 localizes at the bud neck and recruits Rho1 to promote AMR contraction and primary septum formation [8]. As both processes occurred normally in *gps1Δ* cells, we excluded the possibility that Gps1 controls Rho1 by regulating Tus1 (Figure S3). After mitotic exit, the GEF Rom2 controls Rho1 localization [9]. Rom2 localizes at the bud neck after AMR contraction in a manner that is dependent upon phosphatidylinositol 4,5-bisphosphate [28]. However, neither the bud neck localization of Rom2 nor the accumulation of phosphatidylinositol 4,5-bisphosphate at the bud neck was impaired in *gps1Δ* cells (Figures S8 and S9). Furthermore, constitutive targeting of either Rom2 or other Rho GEFs to the bud neck failed to rescue the growth phenotype of *gps1Δ* cells (Figure S9). This reinforces the view that Gps1 promotes Rho1 bud neck targeting in a Rom2-independent manner.

Given that Gps1 did not promote Rho1 GEF localization, we asked whether Gps1 could negatively influence Rho1 GTPase-activating proteins (GAPs). Interestingly, deletion of *LRG1*, but not of other Rho GAP genes, rescued the growth defect of *gps1Δ* cells (Figures 3I and S10A). Since Lrg1 levels at the bud neck were not altered in *gps1Δ* cells (Figure S10B and S10C), we concluded that, although Rho1 was transiently recruited to the bud neck in both *gps1Δ* and *gps1Δ lrg1Δ* cells, deletion of *LRG1* led to the accumulation of higher amounts of active Rho1 that compensated for its function in secondary septum formation. In agreement with this hypothesis, the deletion of *LRG1* could negate the need for *GPS1* in secondary septum formation, most likely because of the increased targeting of Rho1 effectors to the bud neck (Figure 3C, 3H, and 3I). However, since *LRG1* deletion in *gps1Δ* cells did not completely restore Rho1 effectors at the bud neck to the same levels observed in wild-type cells (Figure 3C), we consider it unlikely that Gps1 regulates Rho1 exclusively through inhibition of Lrg1.

Taken together, our data indicate that Gps1 regulates Rho1 bud neck localization by a mechanism that is independent of Rho1 GAP or GEF bud neck targeting.

### Gps1 Regulates Cdc42 Localization after AMR Contraction

Analysis of *gps1Δ* cells by live cell imaging revealed a high percentage of daughter cells (30%–40%) that lysed shortly after AMR contraction and, consequently, were of dark appearance and stained by the cell death marker propidium iodide (Figure S11; Video S3). Because more than 95% of *gps1Δ* cells had a defect in secondary septum formation, we reasoned that daughter cell death could not solely arise from alterations in secondary septum assembly. Given that Cdc42 interacted with Gps1 (Figure 1B–1E), we pursued the idea that daughter cell death in *gps1Δ* cells could be a consequence of mis-regulation of Cdc42.

**Figure 4:**
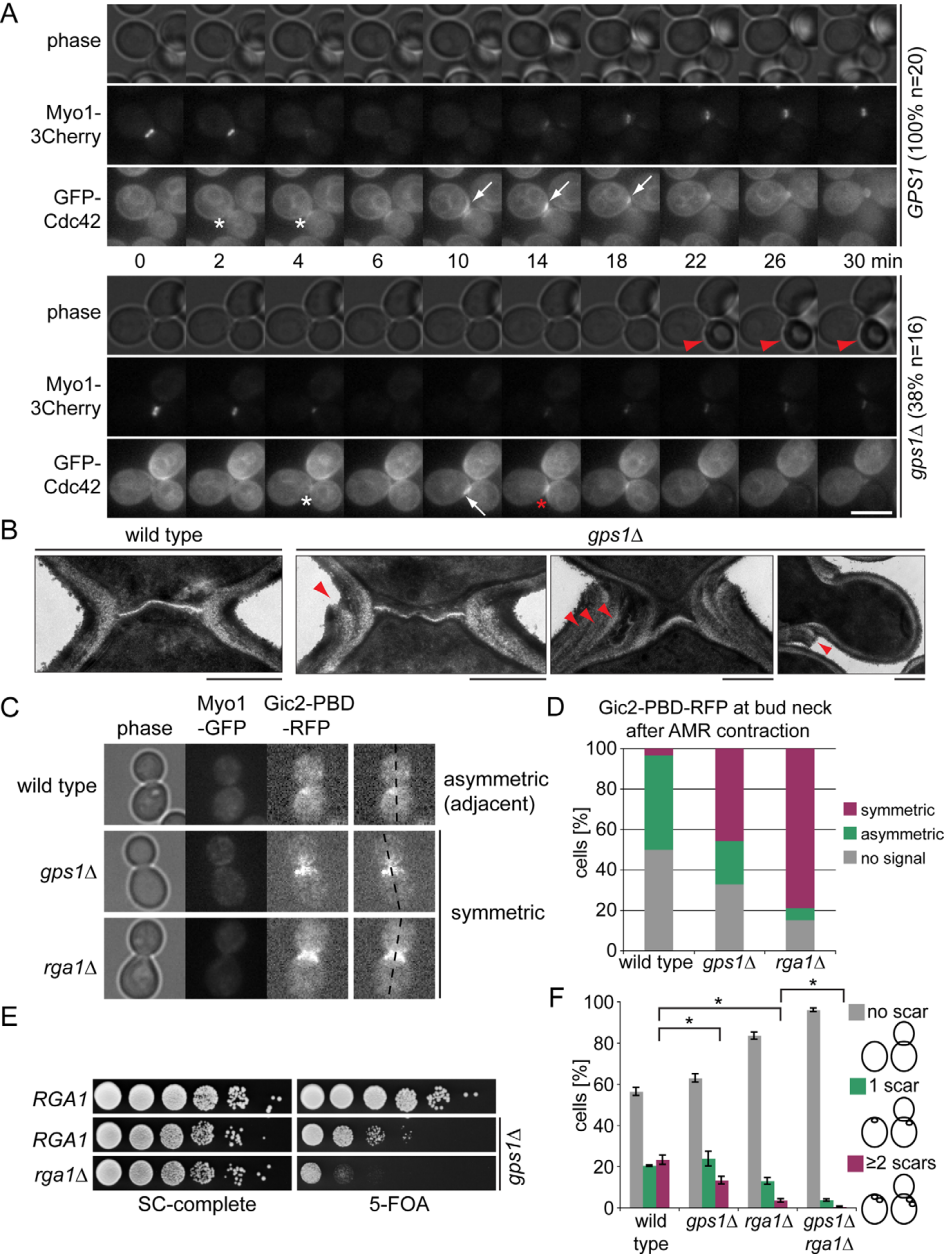
Gps1 regulates Cdc42 localization at the cell division site after AMR contraction. (A) Time-lapse series of GFP-Cdc42 and Myo1-3Cherry at the bud neck in *gps1Δ* and wild-type cells. White asterisk, bud neck localization of Cdc42; white arrow, shift of Cdc42 to the side of the bud neck; red asterisk, back-shift of Cdc42 to the bud neck; red arrowhead, dead daughter cell. The percentage of wild-type and *gps1Δ* cells showing the depicted phenotype is indicated. Scale bar: 5 µm. (B) Electron micrographs of the bud neck region of *gps1Δ* and wild-type cells. Red arrowheads point to additional cell wall collars. Scale bar: 0.5 µm. (C) Symmetric and asymmetric accumulation of the in vivo reporter for active Cdc42 (Gic2-PBD-RFP) after AMR contraction in wild-type, *gps1Δ*, and *rga1Δ* cells. (D) Quantification of (C) (*n*>100 per strain). (E) Genetic interaction between *GPS1* and *RGA1*. SC, synthetic complete medium. (F) Quantification of bud scars (*n*>100 per strain). Error bars show the standard deviation. “*” indicates *p*<0.01.

To investigate whether Gps1 controls Cdc42, we compared Cdc42 localization in wild-type and *gps1Δ* cells (Figure 4A; Videos S4 and S5). In wild-type and *gps1Δ* cells, Cdc42 was recruited to the bud neck prior to AMR contraction (Figure 4A, 2–4 min, white asterisk; Videos S4 and S5). However, whereas Cdc42 moved to the new bud site in every wild-type cell (Figure 4A, white arrows), Cdc42 returned to the bud neck in more than 30% of *gps1Δ* cells (Figure 4A, red asterisk). Strikingly, this backward migration of Cdc42 correlated with the death of the daughter cell, as indicated by the shrinkage and darkening of the bud (Figure 4B, red arrowhead; Video S5). Thus, Gps1 inhibits Cdc42-dependent repolarization and budding at the site of former cell division. Consistently, TEM revealed that more than 30% of *gps1Δ* cells underwent budding inside the old bud neck, which was marked by the formation of two or more layers of cell wall material at the bud neck (Figure 4B, additional collars). To test whether active Cdc42 remained associated with the bud neck in the absence of *GPS1*, we made use of the established in vivo marker for active Cdc42 (Gic2-PBD-RFP) [29]. We found that Gic2-PBD-RFP accumulated at the bud neck after AMR contraction in *gps1Δ* but not in wild-type cells, where Gic2-PBD-RFP became concentrated next to the cell division site (Figure 4C and 4D). Furthermore, we observed that the Cdc42-activating GEF, Cdc24, was enriched at the bud neck in *gps1Δ* but not in wild-type cells after AMR contraction (Figure S12). These data therefore suggest that Gps1 constitutes a safeguard mechanism that inhibits Cdc42 function at the bud neck after cytokinesis.

Interestingly, Cdc42 activation at the old cell division site and the “budding inside the old bud neck” phenotype has previously been reported for Rga1-deficient cells [29]. Rga1 acts as a GAP that inactivates Cdc42. Consequently, *RGA1* deletion causes the bud neck accumulation of active Cdc42 (Figure 4C and 4D). We thus postulated that Gps1 may work either together with, or in parallel to, Rga1 to control Cdc42. We took a genetic approach to discriminate between these possibilities. The deletion of *RGA1* alone did not affect cell growth but compromised the viability of *gps1Δ* cells (Figure 4E). Importantly, *RGA1* was the only Rho GTPase GAP gene whose deletion was synthetic lethal with *gps1Δ* (Figure S10A). In comparison to *gps1Δ* and *rga1Δ* single mutants, *gps1Δ rga1Δ* cells showed an enhanced “budding inside the old bud neck” phenotype, as observed by the reduced number of bud scars formed at mother cells (Figure 4F). Thus, our results suggest that Gps1 may work in a parallel mechanism to the Cdc42 GAP Rga1 to regulate Cdc42 function after AMR contraction.

### Gps1 Creates a Cdc42-Related Inhibition Zone around the Cell Division Site

**Figure 5:**
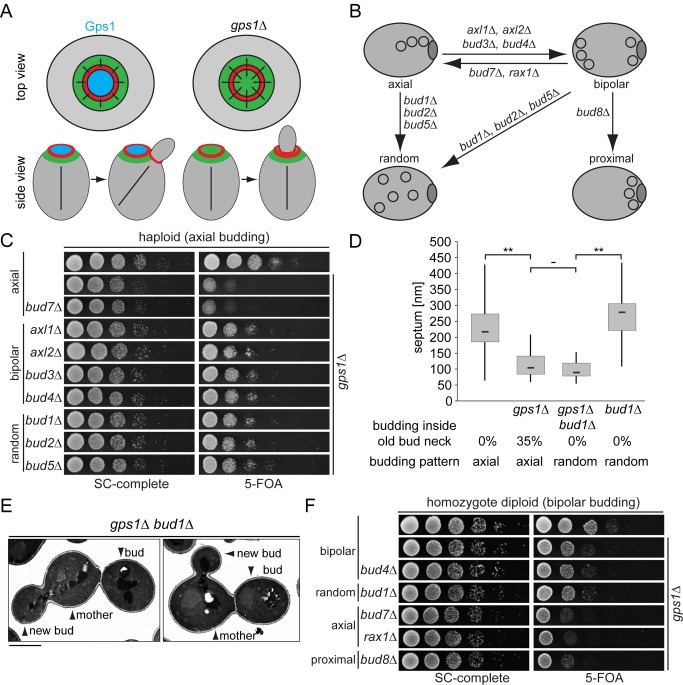
Gps1 creates a Cdc42 inhibition zone around the cell division site. (A) Model for Gps1 function at the bud neck. Red, septin ring with upstream regulators of Cdc42; green, activation area for Cdc42; blue, Gps1 localization creates an inhibition area for Cdc42 activation at the old cell division site. (B) Schematic representation of genes influencing budding pattern and determination of the cell polarity axis after cell division. (C) Growth effect of budding pattern change on haploid *gps1Δ* cells. (D) Quantification of septum thickness and “budding inside the old bud neck” (based on TEM analysis) of wild-type (*n* = 42), *gps1Δ* (*n* = 48), *gps1Δ bud1Δ* (*n* = 30), and *bud1Δ* (*n* = 19) cells. “**” indicates *p*<0.0001; “−” indicates *p*>0.05. (E) Electron micrographs showing randomized budding pattern in *gps1Δ bud1Δ* cells. Scale bar: 2 µm. (F) Growth of diploid *gps1Δ*/*gps1Δ* cells with bipolar, random, axial, or proximal budding pattern as a consequence of the indicated gene deletions. SC, synthetic complete medium.

An important function of Cdc42 is to organize the actin cytoskeleton to allow growth of the new bud. We hypothesized that Gps1 may create an inhibitory zone at the old bud neck to locally impede Cdc42 function after cytokinesis (Figure 5A). Based on the growth pattern of yeast cells, we expected this regulation to be especially important in haploid cells, where the new polarity site is formed adjacent to the old division site (axial budding pattern). In contrast, diploid cells establish a new polarity site distally (bipolar budding pattern). The establishment of the axial polarity site depends on a set of proteins (Bud3, Bud4, Axl1, and Axl2) that localize at the septin rings at the previous division site [30]. To test whether the mode of bud patterning would affect the survival of *gps1Δ* cells, we deleted genes reported to activate axial budding in haploid cells (Figure 5B). The deletion of these genes causes a shift from an axial to a bipolar or random budding pattern in haploid cells [30]. Interestingly, the deletion of the activators of axial budding rescued the growth defect of *gps1Δ* cells (Figure 5C), without affecting secondary septum formation (Figure 5D and 5E). Therefore, we concluded that diminishing the probability of having a new bud site close to the old one increases the viability of *gps1Δ* cells. Conversely, one could imagine that forcing diploid cells to bud axially would have the opposite effect, i.e., would compromise the viability of diploid *gps1Δ/gps1Δ* cells, which were otherwise viable (Figure 5F). As predicted, the viability of diploid cells lacking *GPS1* was significantly reduced after changing the budding pattern from bipolar to axial or proximal (Figure 5F).

Collectively, our data indicate that Gps1 provides an inhibitory signal at the old bud neck that prevents repolarization and rebudding within this area after cytokinesis. This function is important for cell survival.

### Gps1 Regulates Specificity of Cdc42 towards Its Effectors to Facilitate the Establishment of a New Cell Polarity Axis

**Figure 6:**
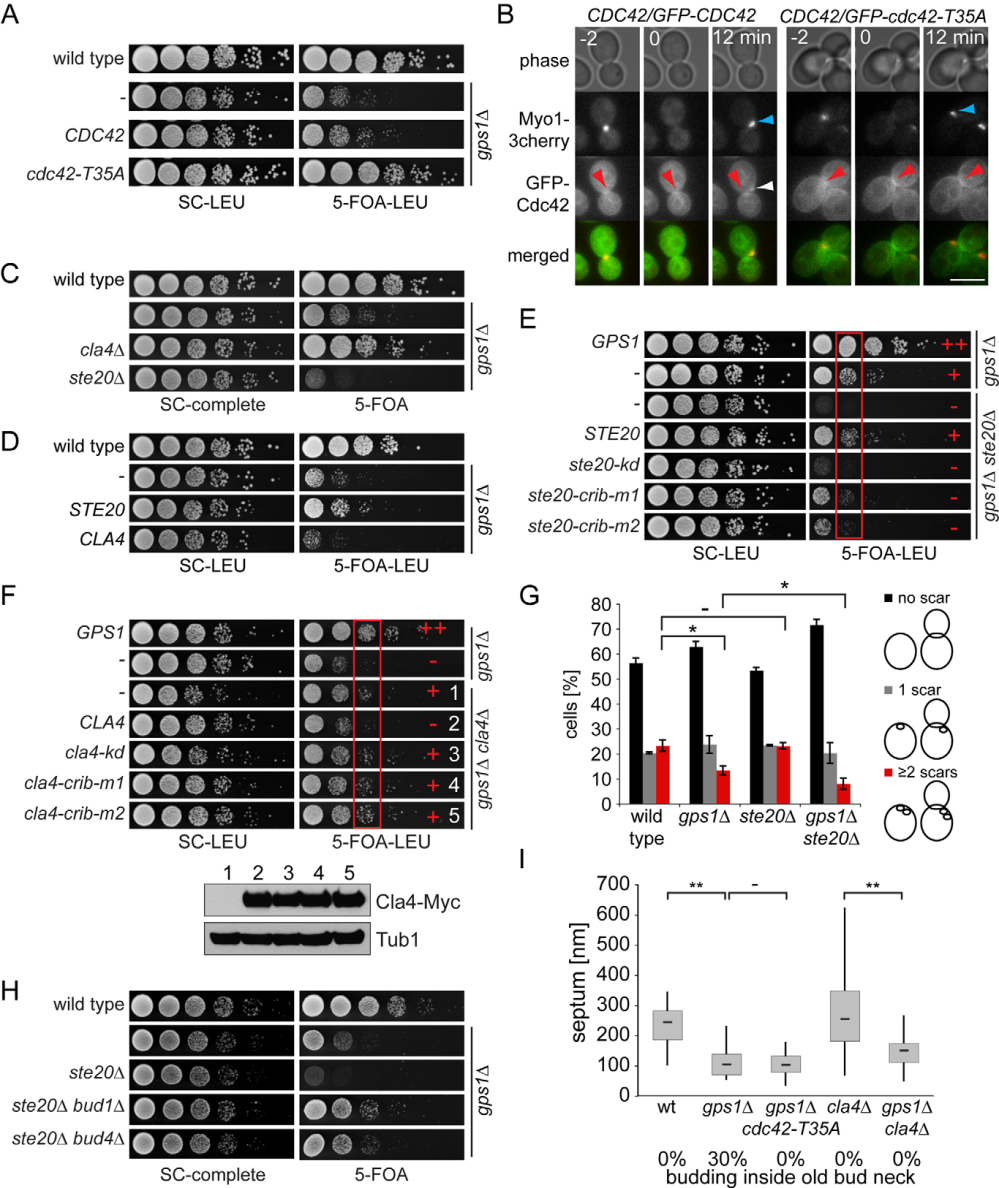
Gps1 regulates Cdc42. (A) Growth effect of the *cdc42-T35A* mutant on *gps1Δ* cells. (B) Bud neck localization of Cdc42-T35A in comparison to wild-type Cdc42. Blue arrowhead, Myo1 polarization next to the old bud neck; white arrowhead, Cdc42 polarization next to the old bud neck; red arrowhead, bud neck localization of Cdc42 and Cdc42-T35A; Scale bar: 5 µm. (C) Genetic interaction of *GPS1* with the Cdc42 effectors *CLA4* and *STE20*. (D) Growth effect of *STE20* and *CLA4* overexpression (2 µ-based plasmid) on *gps1Δ* cells. (E) Genetic interaction between *GPS1* and *STE20* mutants expressed from the low-copy CEN plasmid. *ste20-kd*, Ste20 kinase dead; *ste20-crib-m1* and *ste20-crib-m2*, Ste20 with inactive CRIB-domain. Normal growth (+) and growth sickness or lethality (−) are indicated. The red box depicts the cell culture dilution in which the growth difference between the indicated strains was most pronounced. (F) Genetic interaction between *GPS1* and *CLA4* expressed as in (E). The protein levels of Cla4-3Myc in the indicated strains (1–5) were analyzed by immunoblotting using anti-Myc antibodies. Tub1 served as a loading control. Red box as in (E). (G) Quantification of bud scars (*n*>100 per strain). Error bars show the standard deviation. (H) Genetic lethality of *GPS1* with *STE20* upon *BUD1* or *BUD4* deletion. (I) Quantification of septum thickness and “budding inside the bud neck” phenotype of wild-type (*n* = 34), *gps1Δ* (*n* = 47), *gps1Δ cdc42-T35A* (*n* = 39), *cla4Δ* (*n* = 33), and *gps1Δ cla4Δ* (*n* = 46) cells. “**” indicates *p*<0.0001; “*” indicates *p*<0.01; “−” indicates *p*>0.5. SC, synthetic complete medium.

Cdc42 activates several downstream effectors, including the kinases Cla4 and Ste20. To understand the function of Cdc42 that is regulated by Gps1 at the old bud neck after cytokinesis, we assessed the behavior of established Cdc42 loss-of-function mutants in the *gps1Δ* background. If Gps1 inhibits Cdc42 activity, the down-regulation of Cdc42 function should rescue the growth phenotype of *gps1Δ* cells. In agreement with this hypothesis, a mutation in the effector binding switch I region of Cdc42 (*cdc42-T35A*) rescued the growth defect of *gps1Δ* cells (Figure 6A). This rescue, however, was not consistently supported by locking Cdc42-T35A in the GTP- or GDP-bound forms (Figure S13). Therefore, it is unclear whether the nucleotide-bound state of Cdc42-T35A influences the rescue of *gps1Δ* cells.

To gain a deeper insight onto Cdc42-T35A function in vivo, we monitored its subcellular localization during cytokinesis and polarity establishment in the presence of wild-type Cdc42. Cdc42-T35A was recruited to the bud neck during cytokinesis like wild-type Cdc42 (Figure 6B). However, in contrast to Cdc42, Cdc42-T35A failed to move to the new polarity site after cytokinesis (as judged by the recruitment of Myo1 to the new bud neck) (Figure 6B). Rather, Cdc42-T35A persisted at the old bud neck. Consequently, we suggest that Cdc42-T35A may compete with Cdc42 at the bud neck and probably compensates for the absence of the inhibitory function of Gps1 towards wild-type Cdc42.

Given that Cdc42-T35A has a weaker interaction with effectors, deletion of the gene that encodes the effector protein should also promote the growth of *gps1Δ* cells. Interestingly, the deletion of *CLA4*, but no other Cdc42 effector, improved the growth fitness of *gps1Δ* cells (Figures 6C and S14), suggesting that activation of Cla4 by Cdc42 impairs cell growth in the absence of *GPS1*. Consistently, ectopic overexpression of *CLA4* reduced the viability of *gps1Δ* cells (Figure 6D). Surprisingly, deletion of the Cdc42 effector kinase *STE20* had the opposite impact of *CLA4* deletion, as *gps1Δ ste20Δ* cells were unable to survive (Figure 6C), and overexpression of *STE20* rescued the growth lethality of *gps1Δ* cells (Figure 6D). Importantly, the effect of *CLA4* and *STE20* on the growth of *gps1Δ* cells was dependent upon both the kinase activity and the CRIB-domain that mediates the binding of Cla4 and Ste20 to Cdc42 (Figure 6E and 6F). We therefore concluded that Cdc42-dependent Cla4 activation must be down-regulated, while Cdc42-dependent Ste20 activation must be promoted, to provide viability of cells lacking *GPS1*.

To examine how Cla4 and Ste20 influence the growth of *gps1Δ* cells, we analyzed the phenotype of *gps1Δ ste20Δ* and *gps1Δ cla4Δ* cells in detail. The synthetic defect of *ste20Δ gps1Δ* double mutants was based on an increased “budding inside the old bud neck” phenotype (Figure 6G). Interestingly, Ste20 is required to promote bipolar budding [31], suggesting that increased budding in close proximity to the bud neck may account for the lethality of *ste20Δ gps1Δ* cells. Indeed, the lethality of *ste20Δ gps1Δ* double mutants was reverted upon deletion of *BUD1* or *BUD4* to switch the budding pattern from proximal to bipolar or random (Figure 6H). This suggested that Ste20 activity may be especially important to place the new bud site next to the old cell division site. Analysis of *cdc42-T35A gps1Δ* and *cla4Δ gps1Δ* cells by TEM displayed a much less pronounced “budding inside the old bud neck” phenotype than was observed for *gps1Δ* cells (Figure 6I), indicating that inhibition of Cla4 activity at the bud neck is required to avoid rebudding inside the old bud neck.

Taken together our results indicate that Gps1 inhibits Cdc42-dependent polarity establishment at the old bud neck most likely by promoting Ste20 and inhibiting Cla4 activation.

### Gps1 Prevents Premature Cla4 Activation at the Old Cell Division Site

**Figure 7:**
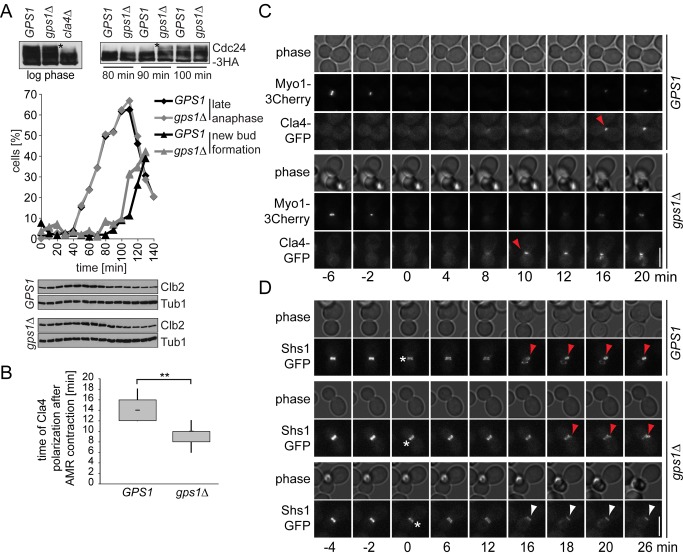
Gps1 inhibits activation of Cdc42-Cla4 at the cell division site. (A) Top left: phosphorylation of Cdc24 in cycling cells of the indicated genotypes; top right: phosphorylation of Cdc24 in wild-type and *gps1Δ* cells arrested in metaphase with nocodazole and released in medium lacking nocodazole. Asterisks indicate Cla4-dependent phosphorylation forms. The graph shows the quantification of anaphase cells (based on segregated DNA masses) and cells with a new emerging bud during the time course (*n*>100 per strain and per time point). The levels of Clb2 are shown as reference for cell cycle progression. Tub1 served as loading control. (B) Time of Cla4 accumulation around the bud region (bud neck and new bud) after AMR contraction was determined for wild-type (*n* = 11) and *gps1Δ* (*n* = 9) cells by time-lapse microscopy using *CLA4-GFP MYO1-3Cherry* cells. “**” indicates *p*<0.0001. (C) Time-lapse series showing the accumulation of Cla4 at or next to the bud neck in *gps1Δ* and wild-type cells. (D) Time-lapse series showing the reorganization of the septin ring (Shs1-GFP) at the bud neck in *gps1Δ* and wild-type cells. White asterisk, septin splitting; red arrowhead, reassembling of a new septin ring next to the old bud neck simultaneous with the disassembling of the septin ring at the mother side of the bud neck; white arrowhead, failure in disassembling of the septin ring, followed by the death of the daughter cell. Scale bars: 5 µm.

To determine whether Gps1 prevents premature activation of Cla4 at the old bud neck, we analyzed the phosphorylation status of Cdc24, a well-known substrate of Cla4 (Figure 7A) [13]. In both wild-type and *gps1Δ* cells, Cla4-dependent hyperphosphorylated forms of Cdc24 were observed (Figure 7A, asterisk), suggesting that hyperphosphorylation of Cdc24 per se was not impaired in the absence of *GPS1*. To investigate the timing of Cdc24 phosphorylation, we transiently arrested wild-type and *gps1Δ* cells in metaphase and followed cell cycle progression and Cdc24 hyperphosphorylation after release from the metaphase block. Both wild-type and *gps1Δ* cells progressed through the cell cycle and completed anaphase with similar kinetics (Figure 7A). However, hyperphosphorylation of Cdc24 and budding were both advanced in *gps1Δ* cells (Figure 7A). This indicates that Cdc24 may prematurely activate Cdc42 in cells lacking *GPS1*. In agreement with this hypothesis, we found premature accumulation of Cla4 at the cell division site in *gps1Δ* mutants (Figures 7B, 7C, and S15).

In addition to Cdc24 activation, Cla4 supports the formation of a stable septin ring at the incipient budding site [32]. To do so, the septin ring has to disassemble at the bud neck after cytokinesis. If Cla4 is prematurely activated at the old bud neck in *gps1Δ* cells, the septin ring should be formed and/or maintained at the old bud neck after cytokinesis. Indeed, whereas the septin ring disappeared from the mother cell bud neck after AMR constriction and reappeared at the incipient bud in wild-type cells (Figure 7D, *GPS1*, red arrowheads), it persisted at the mother cell bud neck of all *gps1Δ* cells in which the daughter cell died after cytokinesis (Figure 7D, *gps1Δ*, white arrowheads). Collectively, our data indicate that Gps1 is essential to keep Cla4 inactive at the old bud neck during cytokinesis.

## Discussion

We understand little about the spatiotemporal coordination of events that follow the contraction of AMR and lead to cell separation and establishment of a new cell polarity axis in budding yeast. The two major Rho GTPases, Cdc42 and Rho1, are recruited to the cell division site prior to the onset of AMR contraction. Rho1 contributes to the formation of the AMR and, subsequently, to secondary septum biogenesis and cell separation. The functions executed by Cdc42 at the cell division site remain to be elucidated. Puzzlingly, Cdc42 plays an important role in the establishment of the axial polarity and bud growth that occurs adjacent to the site of the previous cell division. Although Cdc42 is recruited to the site of cell division prior to AMR contraction, bud growth does not begin at this site during cytokinesis. How this specific Cdc42 function is inhibited during cytokinesis is unknown. Here, we identified the protein Gps1 as a bud neck polarity cue that integrates polarity signaling by maintaining Rho1 and inhibiting Cdc42 at the old cell division site. We propose that the Gps1-mediated mechanism facilitates and safeguards a polarity switch between adjacent sites (old bud neck and new bud neck), thereby coordinating cell division with the establishment of a new cell polarity axis.

We identified Gps1 as a direct interactor of both Rho1 and Cdc42 in the yeast two-hybrid system and established by in vitro and in vivo pull-down experiments that Gps1 is in complex with Rho1 and Cdc42. Live cell imaging of Gps1-GFP revealed an intricate pattern of bud neck recruitment. During AMR constriction and primary septum growth, a new membrane compartment is established by the fusion of membrane material provided by the secretory pathway. Gps1 covered exclusively this emerging disc-like compartment that forms after AMR contraction. Our data show that the Gps1 disc is confined at the bud neck by the splitted septin rings, which act as a diffusion barrier for membrane proteins [18]. Gps1 may then be maintained at the cell division site through a direct or indirect interaction with the membrane or transmembrane protein. Alternatively, Gps1 may associate with the septins via the septin interactors Nap1, Nba1, and Nis1, which were found in the purified Gps1 complex (Figure S1).

Our data are consistent with a role for Gps1 in the coordination of Rho1 and Cdc42 function after AMR contraction. During cytokinesis, actin repolarization towards the bud neck is required for both AMR assembly/contraction and targeted delivery of components involved in septum formation. Rho1, but not Cdc42, plays an important role in actin assembly and vesicle delivery through activation of the formin Bni1 and the exocyst landmark Sec3 at the bud neck. In late anaphase, Rho1 accumulates at the bud neck through its GEF, Tus1. This initial pool of active, GTP-bound Rho1 is required for the completion of AMR assembly and contraction and primary septum formation [8]. Our data now indicate that Tus1 can sustain Rho1 at the bud neck for a very limited period, the time of AMR constriction. The maintenance of Rho1 localization at the bud neck post–AMR contraction was largely dependent upon Gps1. Accordingly, the localization of Rho1 effectors, including Fks1, Bni1, and Sec3, was impaired in cells lacking *GPS1*, which reflected the diminished density of actin cables and the absence of secondary septum formation at the bud neck after AMR contraction in *gps1Δ* cells. Since the localization of other bud-neck-residing Rho1 GEFs such as Rom2 was not impaired, we suggest that Gps1 is directly responsible for the maintenance of Rho1 at the bud neck. Supporting this view, engineered tethering of Rho1 to the bud neck partially relieved the requirement of Gps1 for cytokinesis (Figure 3E and 3F).

**Figure 8:**
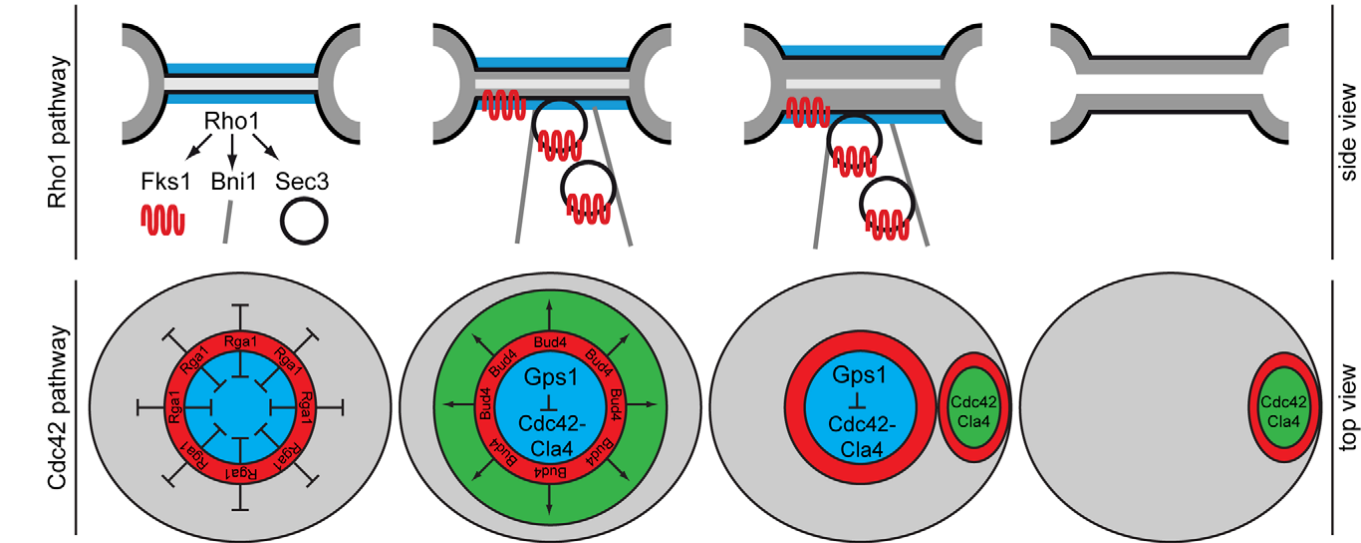
Model for the role of Gps1 in Cdc42 and Rho1 regulation. Top and side views of the bud neck are shown as indicated. See Discussion for details. Red, septin ring with negative (Rga1) and positive (Bud4) upstream regulators of Cdc42; blue, Gps1; green, activation area for Cdc42-Cla4.

In contrast to Rho1, Gps1 inhibited the activation of Cdc42 at the old cell division site after cytokinesis. Consequently, in cells lacking *GPS1*, frequent budding inside the old bud neck was observed by TEM analysis. A similar phenotype has been described for cells bearing hyperactive Cdc42 upon GAP depletion (*rga1Δ* cells) [29]. Rga1 co-localizes with the septin ring that surrounds the cell division site and the Gps1 disc. The synthetic lethal phenotype observed upon co-deletion of *GPS1* and *RGA1* suggests that Gps1 and Rga1 may act in different pathways to inhibit Cdc42. We therefore suggest a model whereby Rga1 regulates the temporal activation of Cdc42, whereas Gps1 dictates directionality by inhibiting Cdc42 at the old cell division site (Figure 8).

Importantly, in contrast to *rga1Δ* cells, *gps1Δ* cells displayed a strong correlation between relocation of Cdc42 to the old bud neck and death of the daughter cell. Genetic manipulations that rescued secondary septum formation improved the growth viability of *gps1Δ* cells without reverting the phenotype of budding inside the old bud neck (Table S1). Conversely, genetic manipulations that decreased the probability of axial budding in haploid cells rescued the growth fitness of *gps1Δ* cells independently of secondary septum biogenesis (Table S1). This indicates that *gps1Δ* cells die as a consequence of two combined defects: formation of a new bud at the old bud neck, and an incorrectly formed secondary septum at that location. We therefore concluded that Gps1 is required for cell viability through the temporal coordination of both the Cdc42 and Rho1 pathways.

Which function of Cdc42 does Gps1 inhibit? Cdc42 and its GEF, Cdc24, associate with the cell division site prior to the onset of AMR contraction. Although the function of Cdc42 at the bud neck at this early stage of cytokinesis is unclear, it is well established that GTP-bound Cdc42 activates several effectors required for polarized growth, including the kinase Cla4. Several lines of evidence indicate that Gps1 inhibits premature Cla4 activation. First, deletion of *CLA4*, but not other Cdc42 effectors, rescued the growth defect caused by deletion of *GPS1*. Second, unlike the normal controls seen in wild-type cells, where Cla4 weakly associated with the bud neck, Cla4 accumulated to high levels at the bud neck and prematurely phosphorylated Cdc24 in *gps1Δ* cells (Figure 7A–7C). Finally, the Cdc42-T35A protein, which fails to activate Cla4 [13] but efficiently localizes at the bud neck (Figure 6B), rescued budding inside the old bud neck and cell viability of *gps1Δ* cells. Thus, we postulate that Gps1 specifically inhibits Cdc42-dependent Cla4 activation at the old bud neck, thereby supporting budding outside the area where cytokinesis takes place. Why should a cell prevent budding inside the old bud scars? First, budding inside the old site of cell division makes cells more vulnerable to defects in secondary septum biogenesis, which may be inheritable (as in case of *GPS1* knock-out) or induced by drugs/toxins that affect cell wall formation. Second, electron micrographs show that recurrent budding at the same position narrows the diameter of the bud neck (Figure 4B). A narrower bud neck may hinder chromosome segregation or partitioning of organelles between mother and daughter cells. In either case, budding inside the old site of cell division increases the probability of cell death.

Cell cycle progression is mainly regulated by differential gene expression, post-translational modifications, and protein degradation. In *Saccharomyces cerevisiae*, the hallmark of the mitotic exit is the activation of the phosphatase Cdc14, which drives the M–G1 phase transition and is a prerequisite for cytokinesis. Cdc14 counteracts mitotic Cdk1, thereby activating the transcription factors Ace2 and Swi5 [24]. Ace2 is well known for its cytokinesis-related function in activating the daughter-specific expression of the chitinase and glucanases that are responsible for the final cleavage of the daughter from the mother cell [33]. In contrast to Ace2, a direct role for Swi5 in cytokinesis and cell separation has remained obscure to date. We showed that Swi5 is essential for Gps1 expression and consequently also for secondary septum formation (Figure 2E). This dependency can be overcome by expressing *GPS1* under the control of a Swi5-independent promoter. The finding that Swi5 regulates secondary septum formation, whereas Ace2 is essential for the following cell cleavage, raises the question whether there is a cross-talk between Swi5- and Ace2-dependent activation to ensure the correct order of function of the Swi5- and Ace2-dependent targets.

In conclusion, the analysis of Gps1 function presented here has provided novel insights into the spatiotemporal coordination of polarity during the late cytokinetic and early morphogenetic events that are under control of Rho1 and Cdc42 signaling. We anticipate that similar mechanisms might coordinate the final stages of division and the reestablishment of cell polarity in other systems in which membrane-associated polarity cues act as a Rho GTPase positioning system and/or promote Rho GTPase activation and inactivation to ensure either a switch-like change of polarity or the maintenance of a stable polarity gradient that is characterized by a spatial separation of Rho GTPase activity.

## Materials and Methods

### Yeast Manipulations and Protein Methods

Yeast strains and plasmids used in this study are listed in Tables S2 and S3. *CDC42* and *RHO1* constructs were expressed from their endogenous promoter. Yeast growth and media were as described previously [34]. Gene deletions and epitope tagging were performed using PCR-based methods [35],[36]. Yeast strains were grown in yeast peptone dextrose medium containing 0.1 mg/l adenine (YPAD). Osmo-sensitive mutants were grown in medium containing 1 M sorbitol for osmo-stabilization. Strains carrying plasmids were grown in synthetic complete medium lacking the corresponding amino acids. For synchronization of cells in the G1 phase, 10 µg/ml synthetic alpha-factor (Sigma-Aldrich) was added to cultures in the early log phase (5×10^6^ cells/ml) and incubated for <2.5 h at 30°C or <4 h at 23°C until >95% of the cells formed mating projections. To arrest the cells with nocodazole, 15 µg/ml nocodazole (Sigma-Aldrich) was added to the culture medium and incubated 2–4 h until >90% of the cells arrested with large buds and one DNA-stained region (DAPI staining). Loss of *URA3*-containing plasmids was assessed by using plates containing 1 mg/ml 5-fluoroorotic acid (5-FOA).

### Genetic Interactions Based on Growth

The plasmid shuffle strategy was used to test the viability of single and double mutants (genetic interaction). Briefly, mutant strains containing the corresponding wild-type gene on an *URA3*-based plasmid (pRS316) were analyzed for growth on 5-FOA plates (which select against *URA3*). At least six individual transformants were analyzed for each mutant, and one representative mutant is shown in Figures 2D, 2I, 2L, 3F, 3I, 4E, 5C, 5F, 6A, 6C–6F, 6H, S4B, S6A, S6B, S6D, S7A, S9A, S10A, S13A, S13B, and S14. For each mutant, 10-fold serial dilutions of wild type (ESM356 or YPH499) and mutants with the indicated genotype were spotted onto synthetic complete medium and 5-FOA plates. Mutants were complemented by pRS316-*HOF1* (Figure 2D) and by pRS316-*GPS1* (Figures 3F, 3I, 4E, 5C, 5F, 6A, 6C–6F, 6H, S4B, S6A, S6B, S6D, S7A, S9A, S10A, S13A, S13B, and S14). Strains carrying genes on a *LEU2*-based plasmid (Figures 6A, 6D–6F, S4B, S6A, S6B, and S13A) were grown on medium lacking leucine to avoid plasmid loss. Plates were incubated for 1 or 2 d at 30°C.

### Protein Detection Methods and Quantifications

Yeast protein extracts and Western blotting were performed as described previously [35]. Antibodies were rabbit anti-GFP antibody, mouse anti-tubulin (Tub1), mouse anti-HA (clone 12CA5, Sigma), mouse anti-Myc (clone 9E10, Sigma), rabbit anti-Clb2, and guinea pig anti-Sic1 [37]. Secondary antibodies were goat anti-mouse, goat anti-rabbit, and goat anti–guinea pig IgGs coupled to horseradish peroxidase (Jackson ImmunoResearch Laboratories).

Slt2 was probed with anti-Mpk1 (y-244, Santa Cruz Biotechnology), and phosphorylated Slt2 was probed with Phospho-p44/42 MAPK (T202/Y204, New England BioLabs). To determine the proportion of activated Slt2, the measured values of phosphorylated and total Slt2 were corrected by subtracting the background signal. Corrected values were plotted as the ratio of phosphorylated Slt2 to total Slt2 (Figure 2H).

### Protein Interaction Assays

#### Immunoprecipitation experiments

Yeast cells were grown until the mid-log phase (10^7^ cells/ml). Pellets from a 100-ml (co-immunoprecipitation experiments) or 1.6-l (large-scale TAP purification) yeast culture were lysed in a FastPrep FP120 Cell Disrupter (MP Biomedicals) using acid-washed glass beads (Sigma-Aldrich). Lysis buffer contained 50 mM Tris-HCl (pH 7.5), 150 mM NaCl, 10% glycerol, 1 mM EDTA, 1 mM DTT, 350 µg/ml benzamidine, 100 mM β-glycerophophate, 50 mM NaF, 5 mM NaVO_3_, and complete EDTA-free protease inhibitor cocktail (Roche). Cell lysates were incubated with 0.5% NP40 for 20 min. Total extracts were clarified by centrifugation at 10,000 *g* for 10 min at 4°C. Gps1-TAP was immunoprecipitated from total extracts using Dynabeads M-270 Epoxy beads (Invitrogen) covalently coupled to rabbit IgG according to manufacturer's instructions. Interacting proteins were identified by mass spectrometry or immunoblotting analysis using specific antibodies.

#### In vitro binding assays

Expression of *GST*, *GST-RHO1*, *GST*-*RHO1-G19V*, *GST*-*RHO1-G22A*, *GST*-*CDC42*, *GST*-*CDC42-D57Y*, *GST*-*CDC42-Q61L*, and *6His*-*GPS1* (amino acids 1–290) was induced in *Escherichia coli* BL21 (DE3) at 30°C. Fusion proteins were purified according to the manufacturers' instructions (GE Healthcare, Qiagen). Purified 6His-Gps1-1-290 bound to Ni^2+^-NTA agarose beads (Qiagen) was washed three times with binding buffer (50 mM Tris-HCl [pH 7.4], 10% glycerol, 100 mM NaCl, 1 mM EDTA, 1 mM DTT, and 0.5% NP-40) and incubated with GST or GST fusion proteins in binding buffer for 1 h at 4°C. The beads were washed five times with binding buffer and analyzed by SDS-PAGE and Western blotting.

#### Yeast two-hybrid system

Indicated genes or gene fragments were cloned into pMM5 and pMM6. Plasmids were transformed into SGY37 and YPH500, and protein interactions were determined as described previously [38].

### Microscopic Techniques and Flow Cytometry

For fluorescence still image analysis, cells carrying GFP or Cherry fusion proteins were fixed in 4% formaldehyde for 10–30 min before inspection. Cells were fixed with 70% ethanol and re-suspended in PBS containing 1 µg/ml DAPI (4′,6-diamino-2-phenylindole, Sigma) for DNA visualization (DAPI staining). For actin staining, cells were fixed for 60 min in 4% formaldehyde solution in PBS. Actin and bud scars were stained with 2 µM rhodamine-phalloidine (Invitrogen) and 0.1 mg/ml calcofluor (Fluorescence Brightener 28, Aldrich-Sigma), respectively. Dead cells were stained with 5 µg/ml propidium iodide. Live cell imaging and quantification of fluorescence still images were performed as described previously [20]. For quantification and image preparation, 3–5 z-sections of the region of interest were averaged or maximum-projected, respectively. Specimens for electron microscopy were prepared as described previously [20]. Flow cytometry was performed as described previously [39].

### Quantification of Bud Scars and PIP2 Levels at the Bud Neck

Bud scars were stained with Fluorescence Brightener 28 (calcofluor, Sigma-Aldrich). Cells were assigned to one of three categories: cells without any bud scar (daughter cells), cells with one bud scar (mother cells), and cells with two or more bud scars (mother cells).

PIP2 (phosphatidylinositol 4,5-bisphosphate) levels were determined using the established PIP2 reporter GFP-2xPH(PLC∂) [40]. To quantify the bud-neck-associated levels of the PIP2 reporter GFP-2xPH(PLC∂), grey values were measured along the mother–daughter axis of still images, as depicted in Figure S8B. PIP2 levels were measured in cells with a large bud after AMR contraction (Figure S8C and S8D) or during cytokinesis (Figure S8E–S8H). For normalization, the level of PIP2 associated with the mother cell membrane was set to 1 in each cell analyzed.

## Supporting Information

Figure S1 **Localization and interaction of Cdc42, Rho1, and Gps1.** (A and B) Localization of GFP-Cdc42 (A) and GFP-Rho1 (B) during different cytokinesis stages at the bud neck in cells expressing *MYO1-3Cherry*. Right panels show enlargements of the depicted areas. (C) List of putative Gps1-TAP interaction partners as identified by mass spectrometry. (D) Time-lapse series show the bud neck localization of Gps1 and the septin Shs1 during cytokinesis. The white arrowhead points toward the new polarity site (new bud neck), where the septin complex assembles. Note that Gps1 does not co-localize with the septin complex at this area (new bud neck).
(TIF)

Figure S2 **Gps1 interaction with Rho1 and Cdc42 is independent of the nucleotide-bound form of the GTPases.** (A) Yeast two-hybrid interactions of Gps1 and Gps1-1-290 with Cdc42, Cdc42-G12V, Cdc42-Q61L, and Cdc42-D57Y. (B) Yeast two-hybrid interactions of Gps1 and Gps1-1-290 with Rho1, Rho1-G19V, Rho1-Q68H, Rho1-C25A, Rho1-F35L, and Rho1-G22A. (C) In vitro binding of 6His-Gps1-1-290 with GST-Rho1, GST-Rho1-G19V, GST-Rho1-G22A, GST-Cdc42, GST-Cdc42-Q61L, and GST-Cdc42-D57Y.
(TIF)

Figure S3 **Gps1 is not required for actomyosin ring contraction and primary septum formation.** (A and B) The timing of AMR contraction in wild-type (wt; *n* = 6) and *gps1Δ* (*n* = 15) cells with respect to spindle break down (*t* = 0) was determined by time-lapse microscopy. (C) Time-lapse series show the recruitment of Chs2-GFP to the bud neck in wild-type and *gps1Δ* cells. Only magnifications of the bud neck region are shown. (D) Quantification of Chs2-GFP relative fluorescence intensity (in arbitrary units) at the bud neck of wild-type (*n* = 4) and *gps1Δ* (*n* = 4) cells. Error bars show the standard deviation.
(TIF)

Figure S4 **Functionality of GFP-Rho1.** (A) Ectopic expression of *RHO1* (CEN plasmid), *GFP-RHO1* (CEN and 2 µ plasmid) and GFP-RHO1* (CEN plasmid; derived from SP301) [9] in a yeast strain where the endogenous *RHO1* is under the control of a glucose-mediated repressible pGAL1 promoter. Higher concentrations of *GFP-RHO1* can partially substitute wild-type *RHO1*. (B) Artificial targeting of GFP-Rho1 or GFP-Rho1* to the cell division site can partially rescue the growth defect of *gps1Δ* cells (see also Figures 3 and S6). Shown are serial dilutions of yeast strains with the indicated genotypes. All strains contain a *URA3/CEN* plasmid harboring wild-type *GPS1*. Cells growing on synthetic complete medium–LEU are able to keep the *URA3-GPS1* plasmid, whereas 5-FOA-LEU selects against the *URA3*-containing plasmid.
(TIF)

Figure S5 **Gps1 is required to maintain Rho1-dependent polarization at the bud neck after actomyosin ring contraction.** (A and B) Time-lapse series show the co-localization of GFP-Fks1 and Myo2-3Cherry at the bud neck during cytokinesis in wild-type (A) and *gps1Δ* (B) cells. (C and D) Quantification of GFP-Fks1 and Myo2-3Cherry at the bud neck in wild-type (*n* = 3) and *gps1Δ* (*n* = 3) cells. Error bars show the standard deviation. (E) The behavior of actin cables (labeled with Abp140-GFP) was analyzed by time-lapse microscopy in wild-type and *gps1Δ* cells. At the first time point, Abp140-GFP localizes at the bud neck as a ring (white arrowhead) (defined as *t* = 0). At later time points, actin cables toward the bud neck are visible in wild-type but not in *gps1Δ* cells. (F) Actin patches and cables were labeled with rhodamine-phalloidine and inspected in large budded *MYO1-GFP* cells (in the presence or absence of *GPS1*) that underwent AMR contraction (absence of Myo1-GFP at the bud neck). The percentage of cells with the indicated pattern of actin cables is indicated (*n* = 150–200 cells per strain). (G) The thickness of the septum (primary septum+secondary septum) of *GPS1* and *gps1Δ* without or with overexpression of *FKS1* (endogenous promoter from a 2 µ-based plasmid, 2 µ-*FKS1*) was quantified by electron microscopy analysis (*GPS1*, *n* = 10; *gps1Δ*, *n* = 14; *gps1Δ* 2 µ-*FKS1*, *n* = 14; *GPS1* 2 µ-*FKS1*, *n* = 35). PS, primary septum; SS, secondary septum. Scale bars: 5 µm.
(TIF)

Figure S6 **Rho1 rescue experiments of** ***gps1Δ*** **cells.** (A) Serial dilutions of the *gps1Δ URA3-GPS1* cells empty or carrying *GPS1*- or *RHO1*-containing low-copy plasmids (*CEN*, *LEU2*). 5-FOA selects against the *URA3*-containing plasmid. (B) Serial dilutions of the *gps1Δ URA3-GPS1* cells carrying an empty or a *RHO1* high-copy plasmid (2 µ, *LEU2*). (C) The thickness of the septum (primary septum+secondary septum) of *gps1Δ GFP-RHO1* (*n* = 25) and *gps1Δ GFP-rho1-C25A* (*n* = 27) was quantified by electron microscopy analysis. “*” indicates *p*<0.01. Scale bars: 5 µm. (D) Serial dilutions of *gps1Δ* cells carrying *GFP-RHO1*, *GFP-rho1-G19V*, *GFP-rho1-Q68H*, *GFP-rho1-C25A*, and *GFP-rho1-F35L* with and without *SHS1-GBP*. (E) Still images show the localization of GFP-Rho1, GFP-Rho1-G19V, GFP-Rho1-Q68H, GFP-Rho1-C25A, and GFP-Rho1-F35L in cells with or without *SHS1-GBP* at different cell cycle stages. (F) Immunoblot shows protein levels of GFP-Rho1, GFP-Rho1-G19V, GFP-Rho1-Q68H, GFP-Rho1-C25A, and GFP-Rho1-F35L with and without *SHS1-GBP*. An unspecific signal of the GFP antibody was used as a loading control. (G) Localization of Sec3-3Cherry and GFP-Rho1 at the bud neck in wild-type, *gps1Δ*, and *gps1Δ SHS1-GBP* cells. (H) Quantification of Sec3-3Cherry in wild-type (*n* = 5), *gps1Δ* (*n* = 5), and *gps1Δ SHS1-GBP* (*n* = 5) cells expressing *GFP-RHO1*. Error bars show the standard deviation.
(TIF)

Figure S7 **Rho2, Rho4, and Cdc42 are not involved in the Gps1-mediated septum formation pathway.** (A) Serial dilutions of *gps1Δ* cells carrying *GFP-CDC42*, *GFP-RHO2*, or *GFP-RHO4* with and without *SHS1-GBP*. (B–D) Still images of *gps1Δ* cells carrying *GFP-CDC42*, *GFP-RHO2*, or *GFP-RHO4* with and without *SHS1-GBP*. (E) Quantification of time-lapse analysis of Sec3-GFP recruitment to the cell division site in wild-type, *rho2Δ*, and *rho4Δ* cells. Error bars show the standard deviation. Scale bar: 5 µm.
(TIF)

Figure S8 **Gps1 is not required for PIP2 accumulation at the bud neck.** (A) PIP2 (stained with GFP-2xPH) concentrates at the bud neck after (top panel) but not before (lower panel) AMR (Myo1) contraction. (B–D) Quantification of PIP2 at the bud neck of wild-type and *gps1Δ* cells. Scheme representation (B) and plot profiles (C and D) of PIP2 (GFP-2xPH fluorescence intensity) at the bud neck in wild-type and *gps1Δ* cells. BN, bud neck region. (E–H) Time-lapse series showing PIP2 (GFP-2xPH) localization during cytokinesis in wild-type (E and F) and *gps1Δ* (G and H) cells. (F) and (H) show the quantification of (E) and (G), respectively. Scale bars: 5 µm.
(TIF)

Figure S9 **Gps1 is not required for Rho1 GEF localization at the bud neck.** (A) Growth of serial dilutions of *gps1Δ* and *gps1Δ SHS1-GBP* cells carrying the Rho1 GEFs Tus1-GFP, Rom1-GFP, or Rom2-GFP. Note that Shs1-GBP tethers the GFP fusion protein to the bud neck constitutively. (B–D) Still images for the Rho1 GEFs Tus1-GFP, Rom1-GFP, or Rom2-GFP in wild-type, *gps1Δ*, and *gps1Δ SHS1-GBP* cells in different cell cycle stages. (E) Time-lapse series are shown for the localization of Rom2-GFP at the bud neck in wild-type, *gps1Δ*, and *gps1Δ SHS1-GBP* cells. Note that Rom2-GFP gives a dim signal at the bud neck in time-lapse analysis (red arrowhead) in both wild-type and *gps1Δ* cells. Scale bars: 5 µm.
(TIF)

Figure S10 **Rho GTPase GAPs and their involvement in the Gps1 pathway.** (A) Growth of serial dilutions of *gps1Δ* cells with and without additional deletion of Rho GTPase GAPs as indicated. (B) Time-lapse series show Lrg1-GFP localization at the cell division site in wild-type and *gps1Δ* cells. (C) Graph shows the quantification of Lrg1-GFP signals at the bud neck in wild-type (*n* = 4) and *gps1Δ* (*n* = 3) cells. Error bars represent the standard deviation.
(TIF)

Figure S11 **Cell death analysis of** ***gps1Δ*** **cells.** Wild-type and *gps1Δ* cells with *MYO1-3Cherry* and *GFP-RHO1* are stained with the cell death marker propidium iodide (PI). Scale bar: 10 µm.
(TIF)

Figure S12 **Cdc24 localization in** ***gps1Δ*** **cells.** Time-lapse series show the localization of Cdc24-GFP in comparison to Myo1-3Cherry in wild-type and *gps1Δ* cells. Cdc24 accumulation at the bud neck (white arrowheads) and adjacent to the bud neck (red arrowheads) is depicted. White asterisks mark a dead daughter cell.
(TIF)

Figure S13 **Cdc42-T35A rescue of** ***gps1Δ*** **is not consistently supported by locking Cdc42 in the GTP- or GDP-bound form.** (A and B) Serial dilutions of *gps1Δ* cells expressing different *CDC42* mutants from a low-copy plasmid (A) or stably integrated into the genome (B). Protein levels are shown. With the exception of GFP-Cdc42-T35A-D118A, the protein amounts of all mutants are comparable to wild-type GFP-Cdc42. Differences between (A) and (B) may result from different expression levels.
(TIF)

Figure S14 **Genetic interaction between** ***GPS1*** **and Cdc42 effectors.** Growth of wild-type and *gps1Δ* cells upon deletion of the indicated Cdc42 effectors.
(TIF)

Figure S15 **Cla4 localization in** ***gps1Δ*** **cells.** (A) Quantification of the total cell signal of Cla4-GFP in a time-lapse series in *GPS1* (*n* = 4) and *gps1Δ* (*n* = 4) cells. Error bars show the standard deviation. (B) Protein amounts of Cla4-GFP in *GPS1* and *gps1Δ* cells determined by immunoblot. An unspecific signal was used as a loading control.
(TIF)

Table S1 **Rescue of** ***gps1Δ*** **defects.**
(DOC)

Table S2 **Yeast strains used in this study.**
(DOC)

Table S3 **Plasmids used in this study.**
(DOC)

Video S1 **Localization of GFP-Rho1 in wild type.** Phase contrast (left), GFP-Rho1 localization (middle), and AMR contraction (right, Myo1-3Cherry) are shown in a representative wild-type cell.
(MOV)

Video S2 **Localization of GFP-Rho1 in** ***gps1Δ*** **.** Phase contrast (left), GFP-Rho1 localization (middle), and AMR contraction (right, Myo1-3Cherry) are shown in a representative *gps1Δ* cell.
(MOV)

Video S3 **Death of the daughter cell in** ***gps1Δ*** **.** Phase contrast (left), GFP-Rho1 localization (middle), and propidium iodide staining as cell death marker (right) are shown in a representative *gps1Δ* cell.
(MOV)

Video S4 **Localization of GFP-Cdc42 in wild type.** Phase contrast (left), GFP-Cdc42 localization (right), and AMR contraction (middle, Myo1-3Cherry) are shown in a representative wild-type cell.
(MOV)

Video S5 **Localization of GFP-Cdc42 in** ***gps1Δ*** **.** Phase contrast (left), GFP-Cdc42 localization (right), and AMR contraction (middle, Myo1-3Cherry) are shown in a representative *gps1Δ* cell.
(MOV)

## Abbreviations

5-FOA: 5-fluoroorotic acid
AMR: actomyosin ring
CWI: cell wall integrity
GAP: GTPase-activating protein
GBP: GFP-binding protein
GEF: guanine nucleotide exchange factor
MAP: mitogen-activated protein
TEM: transmission electron microscopy

## References

[1] Martin-Belmonte F, Perez-Moreno M (2011) Epithelial cell polarity, stem cells and cancer. Nat Rev Cancer 12: 23–38. 22169974 10.1038/nrc3169

[2] McCaffrey LM, Macara IG (2009) Widely conserved signaling pathways in the establishment of cell polarity. Cold Spring Harb Perspect Biol 1: a001370. 20066082 10.1101/cshperspect.a001370 PMC2742088

[3] Park HO, Bi E (2007) Central roles of small GTPases in the development of cell polarity in yeast and beyond. Microbiol Mol Biol Rev 71: 48–96. 17347519 10.1128/MMBR.00028-06 PMC1847380

[4] Iden S, Collard JG (2008) Crosstalk between small GTPases and polarity proteins in cell polarization. Nat Rev Mol Cell Biol 9: 846–859. 18946474 10.1038/nrm2521

[5] Benink HA, Bement WM (2005) Concentric zones of active RhoA and Cdc42 around single cell wounds. J Cell Biol 168: 429–439. 15684032 10.1083/jcb.200411109 PMC2171735

[6] Nelson WJ (2009) Remodeling epithelial cell organization: transitions between front-rear and apical-basal polarity. Cold Spring Harb Perspect Biol 1: a000513. 20066074 10.1101/cshperspect.a000513 PMC2742086

[7] Tolliday N, VerPlank L, Li R (2002) Rho1 directs formin-mediated actin ring assembly during budding yeast cytokinesis. Curr Biol 12: 1864–1870. 12419188 10.1016/s0960-9822(02)01238-1

[8] Yoshida S, Kono K, Lowery DM, Bartolini S, Yaffe MB, et al (2006) Polo-like kinase Cdc5 controls the local activation of Rho1 to promote cytokinesis. Science 313: 108–111. 16763112 10.1126/science.1126747

[9] Yoshida S, Bartolini S, Pellman D (2009) Mechanisms for concentrating Rho1 during cytokinesis. Genes Dev 23: 810–823. 19339687 10.1101/gad.1785209 PMC2666341

[10] Kohno H, Tanaka K, Mino A, Umikawa M, Imamura H, et al (1996) Bni1p implicated in cytoskeletal control is a putative target of Rho1p small GTP binding protein in Saccharomyces cerevisiae. EMBO J 15: 6060–6068. 8947028 PMC452427

[11] Guo W, Tamanoi F, Novick P (2001) Spatial regulation of the exocyst complex by Rho1 GTPase. Nat Cell Biol 3: 353–360. 11283608 10.1038/35070029

[12] Qadota H, Python CP, Inoue SB, Arisawa M, Anraku Y, et al (1996) Identification of yeast Rho1p GTPase as a regulatory subunit of 1,3-beta-glucan synthase. Science 272: 279–281. 8602515 10.1126/science.272.5259.279

[13] Gulli MP, Jaquenoud M, Shimada Y, Niederhauser G, Wiget P, et al (2000) Phosphorylation of the Cdc42 exchange factor Cdc24 by the PAK-like kinase Cla4 may regulate polarized growth in yeast. Mol Cell 6: 1155–1167. 11106754 10.1016/s1097-2765(00)00113-1

[14] Park HO, Bi E, Pringle JR, Herskowitz I (1997) Two active states of the Ras-related Bud1/Rsr1 protein bind to different effectors to determine yeast cell polarity. Proc Natl Acad Sci U S A 94: 4463–4468. 9114012 10.1073/pnas.94.9.4463 PMC20745

[15] Irazoqui JE, Gladfelter AS, Lew DJ (2003) Scaffold-mediated symmetry breaking by Cdc42p. Nat Cell Biol 5: 1062–1070. 14625559 10.1038/ncb1068

[16] Kozubowski L, Saito K, Johnson JM, Howell AS, Zyla TR, et al (2008) Symmetry-breaking polarization driven by a Cdc42p GEF-PAK complex. Curr Biol 18: 1719–1726. 19013066 10.1016/j.cub.2008.09.060 PMC2803100

[17] Huh WK, Falvo JV, Gerke LC, Carroll AS, Howson RW, et al (2003) Global analysis of protein localization in budding yeast. Nature 425: 686–691. 14562095 10.1038/nature02026

[18] Dobbelaere J, Barral Y (2004) Spatial coordination of cytokinetic events by compartmentalization of the cell cortex. Science 305: 393–396. 15256669 10.1126/science.1099892

[19] Meitinger F, Petrova B, Lombardi IM, Bertazzi DT, Hub B, et al (2010) Targeted localization of Inn1, Cyk3 and Chs2 by the mitotic-exit network regulates cytokinesis in budding yeast. J Cell Sci 123: 1851–1861. 20442249 10.1242/jcs.063891

[20] Meitinger F, Boehm ME, Hofmann A, Hub B, Zentgraf H, et al (2011) Phosphorylation-dependent regulation of the F-BAR protein Hof1 during cytokinesis. Genes Dev 25: 875–888. 21498574 10.1101/gad.622411 PMC3078711

[21] Levin DE (2011) Regulation of cell wall biogenesis in Saccharomyces cerevisiae: the cell wall integrity signaling pathway. Genetics 189: 1145–1175. 22174182 10.1534/genetics.111.128264 PMC3241422

[22] Lagorce A, Hauser NC, Labourdette D, Rodriguez C, Martin-Yken H, et al (2003) Genome-wide analysis of the response to cell wall mutations in the yeast Saccharomyces cerevisiae. J Biol Chem 278: 20345–20357. 12644457 10.1074/jbc.M211604200

[23] Doolin MT, Johnson AL, Johnston LH, Butler G (2001) Overlapping and distinct roles of the duplicated yeast transcription factors Ace2p and Swi5p. Mol Microbiol 40: 422–432. 11309124 10.1046/j.1365-2958.2001.02388.x

[24] Visintin R, Craig K, Hwang ES, Prinz S, Tyers M, et al (1998) The phosphatase Cdc14 triggers mitotic exit by reversal of Cdk-dependent phosphorylation. Mol Cell 2: 709–718. 9885559 10.1016/s1097-2765(00)80286-5

[25] Toyn JH, Johnson AL, Donovan JD, Toone WM, Johnston LH (1997) The Swi5 transcription factor of Saccharomyces cerevisiae has a role in exit from mitosis through induction of the cdk-inhibitor Sic1 in telophase. Genetics 145: 85–96. 9017392 10.1093/genetics/145.1.85 PMC1207787

[26] Bertazzi DT, Kurtulmus B, Pereira G (2011) The cortical protein Lte1 promotes mitotic exit by inhibiting the spindle position checkpoint kinase Kin4. J Cell Biol 193: 1033–1048. 21670215 10.1083/jcb.201101056 PMC3115795

[27] Delley PA, Hall MN (1999) Cell wall stress depolarizes cell growth via hyperactivation of RHO1. J Cell Biol 147: 163–174. 10508863 10.1083/jcb.147.1.163 PMC2164985

[28] Audhya A, Emr SD (2002) Stt4 PI 4-kinase localizes to the plasma membrane and functions in the Pkc1-mediated MAP kinase cascade. Dev Cell 2: 593–605. 12015967 10.1016/s1534-5807(02)00168-5

[29] Tong Z, Gao XD, Howell AS, Bose I, Lew DJ, et al (2007) Adjacent positioning of cellular structures enabled by a Cdc42 GTPase-activating protein-mediated zone of inhibition. J Cell Biol 179: 1375–1384. 18166650 10.1083/jcb.200705160 PMC2373499

[30] Casamayor A, Snyder M (2002) Bud-site selection and cell polarity in budding yeast. Curr Opin Microbiol 5: 179–186. 11934615 10.1016/s1369-5274(02)00300-4

[31] Ni L, Snyder M (2001) A genomic study of the bipolar bud site selection pattern in Saccharomyces cerevisiae. Mol Biol Cell 12: 2147–2170. 11452010 10.1091/mbc.12.7.2147 PMC55669

[32] Versele M, Thorner J (2004) Septin collar formation in budding yeast requires GTP binding and direct phosphorylation by the PAK, Cla4. J Cell Biol 164: 701–715. 14993234 10.1083/jcb.200312070 PMC2172161

[33] Colman-Lerner A, Chin TE, Brent R (2001) Yeast Cbk1 and Mob2 activate daughter-specific genetic programs to induce asymmetric cell fates. Cell 107: 739–750. 11747810 10.1016/s0092-8674(01)00596-7

[34] Sherman F (1991) Getting started with yeast. Methods Enzymol 194: 3–21. 2005794 10.1016/0076-6879(91)94004-v

[35] Janke C, Magiera MM, Rathfelder N, Taxis C, Reber S, et al (2004) A versatile toolbox for PCR-based tagging of yeast genes: new fluorescent proteins, more markers and promoter substitution cassettes. Yeast 21: 947–962. 15334558 10.1002/yea.1142

[36] Knop M, Siegers K, Pereira G, Zachariae W, Winsor B, et al (1999) Epitope tagging of yeast genes using a PCR-based strategy: more tags and improved practical routines. Yeast 15: 963–972. 10407276 10.1002/(SICI)1097-0061(199907)15:10B<963::AID-YEA399>3.0.CO;2-W

[37] Maekawa H, Priest C, Lechner J, Pereira G, Schiebel E (2007) The yeast centrosome translates the positional information of the anaphase spindle into a cell cycle signal. J Cell Biol 179: 423–436. 17967947 10.1083/jcb.200705197 PMC2064790

[38] Palani S, Meitinger F, Boehm ME, Lehmann WD, Pereira G (2012) Cdc14-dependent dephosphorylation of Inn1 contributes to Inn1-Cyk3 complex formation. J Cell Sci 125: 3091–3096. 22454527 10.1242/jcs.106021

[39] Geil C, Schwab M, Seufert W (2008) A nucleolus-localized activator of Cdc14 phosphatase supports rDNA segregation in yeast mitosis. Curr Biol 18: 1001–1005. 18595708 10.1016/j.cub.2008.06.025

[40] Stefan CJ, Audhya A, Emr SD (2002) The yeast synaptojanin-like proteins control the cellular distribution of phosphatidylinositol (4,5)-bisphosphate. Mol Biol Cell 13: 542–557. 11854411 10.1091/mbc.01-10-0476 PMC65648

