# Supplementary figures and images for: A Safeguard Mechanism Regulates Rho GTPases to Coordinate Cytokinesis with the Establishment of Cell Polarity
Source: PLoS Biol. 2013 Feb 26;11(2):e1001495. doi: 10.1371/journal.pbio.1001495 (PMC3582507; doi:10.1371/journal.pbio.1001495)

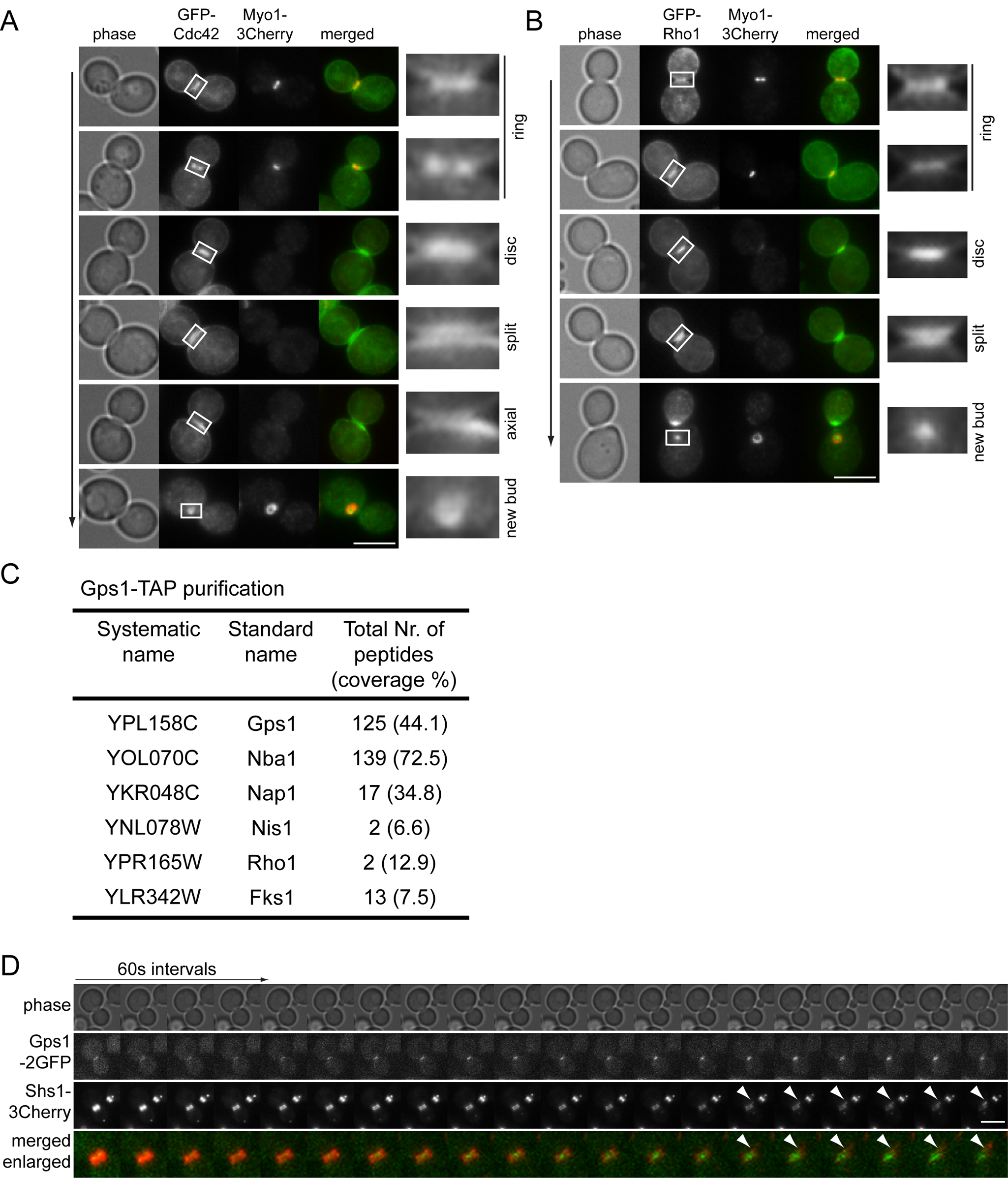

Supplement: Figure S1 — Localization and interaction of Cdc42, Rho1, and Gps1. (A and B) Localization of GFP-Cdc42 (A) and GFP-Rho1 (B) during different cytokinesis stages at the bud neck in cells expressing MYO1-3Cherry. Right panels show enlargements of the depicted areas. (C) List of putative Gps1-TAP interaction partners as identified by mass spectrometry. (D) Time-lapse series show the bud neck localization of Gps1 and the septin Shs1 during cytokinesis. The white arrowhead points toward the new polarity site (new bud neck), where the septin complex assembles. Note that Gps1 does not co-localize with the septin complex at this area (new bud neck). (TIF) [file pbio.1001495.s001.tif]

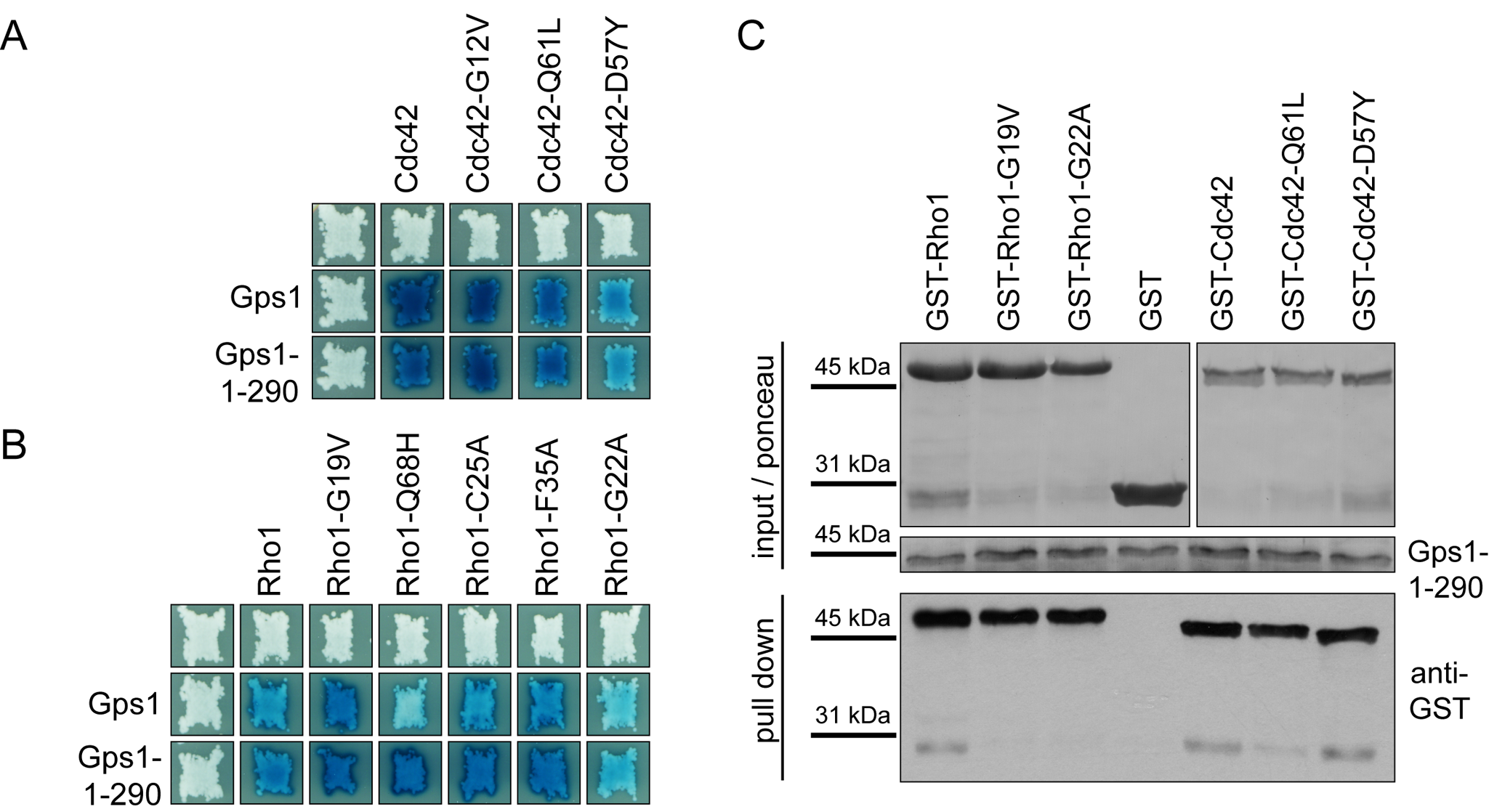

Supplement: Figure S2 — Gps1 interaction with Rho1 and Cdc42 is independent of the nucleotide-bound form of the GTPases. (A) Yeast two-hybrid interactions of Gps1 and Gps1-1-290 with Cdc42, Cdc42-G12V, Cdc42-Q61L, and Cdc42-D57Y. (B) Yeast two-hybrid interactions of Gps1 and Gps1-1-290 with Rho1, Rho1-G19V, Rho1-Q68H, Rho1-C25A, Rho1-F35L, and Rho1-G22A. (C) In vitro binding of 6His-Gps1-1-290 with GST-Rho1, GST-Rho1-G19V, GST-Rho1-G22A, GST-Cdc42, GST-Cdc42-Q61L, and GST-Cdc42-D57Y. (TIF) [file pbio.1001495.s002.tif]

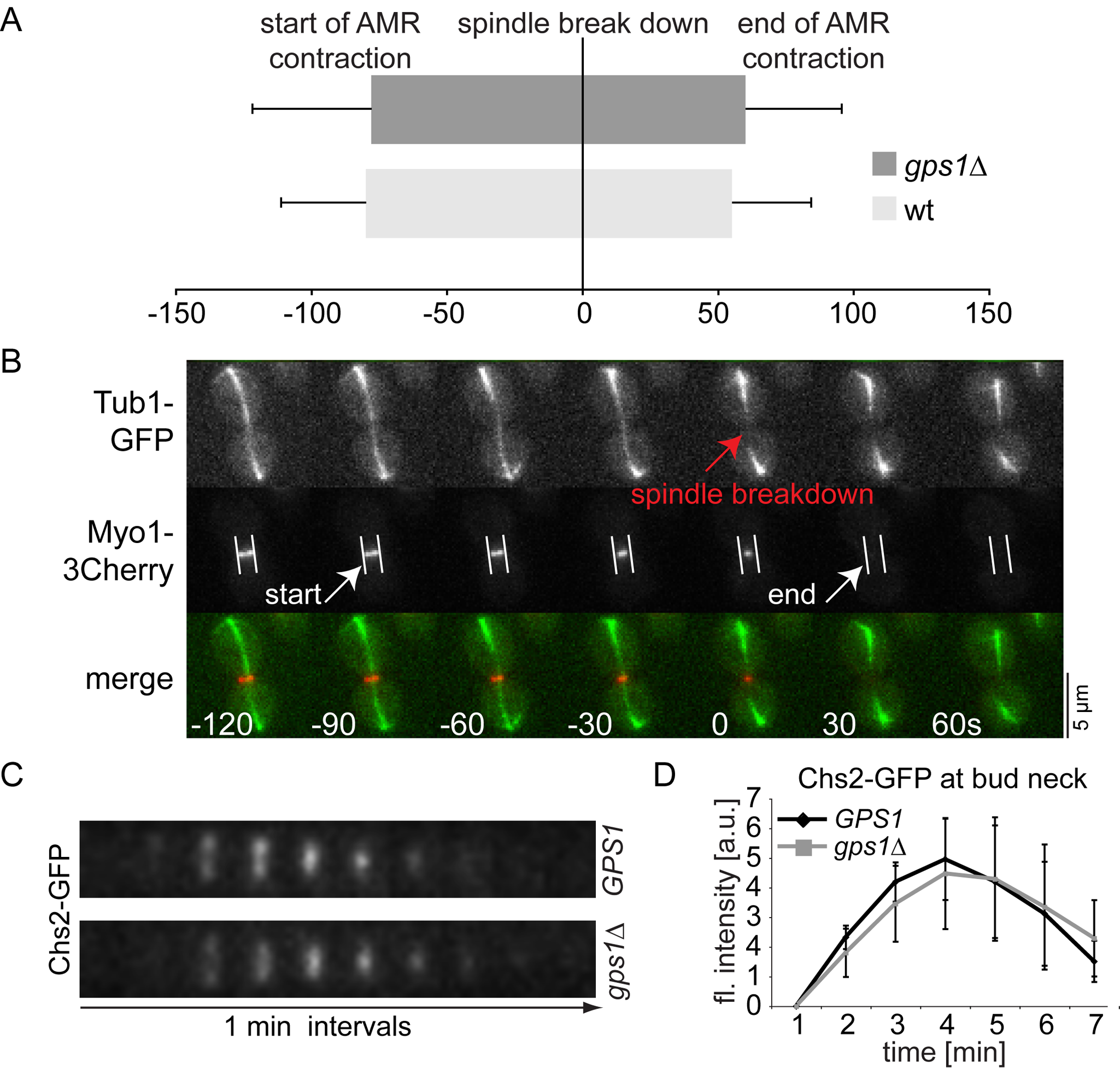

Supplement: Figure S3 — Gps1 is not required for actomyosin ring contraction and primary septum formation. (A and B) The timing of AMR contraction in wild-type (wt; n = 6) and gps1Δ (n = 15) cells with respect to spindle break down (t = 0) was determined by time-lapse microscopy. (C) Time-lapse series show the recruitment of Chs2-GFP to the bud neck in wild-type and gps1Δ cells. Only magnifications of the bud neck region are shown. (D) Quantification of Chs2-GFP relative fluorescence intensity (in arbitrary units) at the bud neck of wild-type (n = 4) and gps1Δ (n = 4) cells. Error bars show the standard deviation. (TIF) [file pbio.1001495.s003.tif]

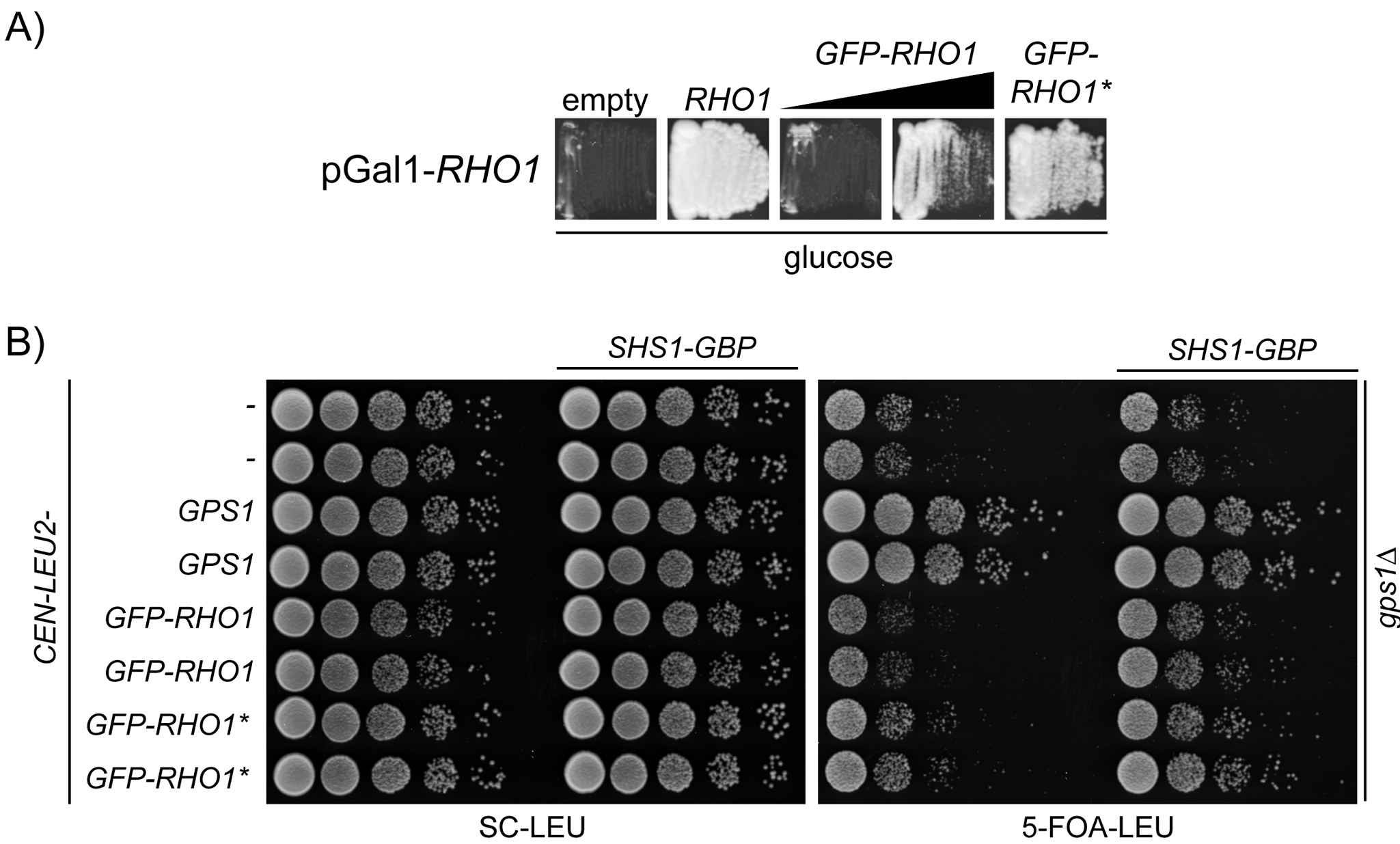

Supplement: Figure S4 — Functionality of GFP-Rho1. (A) Ectopic expression of RHO1 (CEN plasmid), GFP-RHO1 (CEN and 2 µ plasmid) and GFP-RHO1* (CEN plasmid; derived from SP301) [9] in a yeast strain where the endogenous RHO1 is under the control of a glucose-mediated repressible pGAL1 promoter. Higher concentrations of GFP-RHO1 can partially substitute wild-type RHO1. (B) Artificial targeting of GFP-Rho1 or GFP-Rho1* to the cell division site can partially rescue the growth defect of gps1Δ cells (see also Figures 3 and S6). Shown are serial dilutions of yeast strains with the indicated genotypes. All strains contain a URA3/CEN plasmid harboring wild-type GPS1. Cells growing on synthetic complete medium–LEU are able to keep the URA3-GPS1 plasmid, whereas 5-FOA-LEU selects against the URA3-containing plasmid. (TIF) [file pbio.1001495.s004.tif]

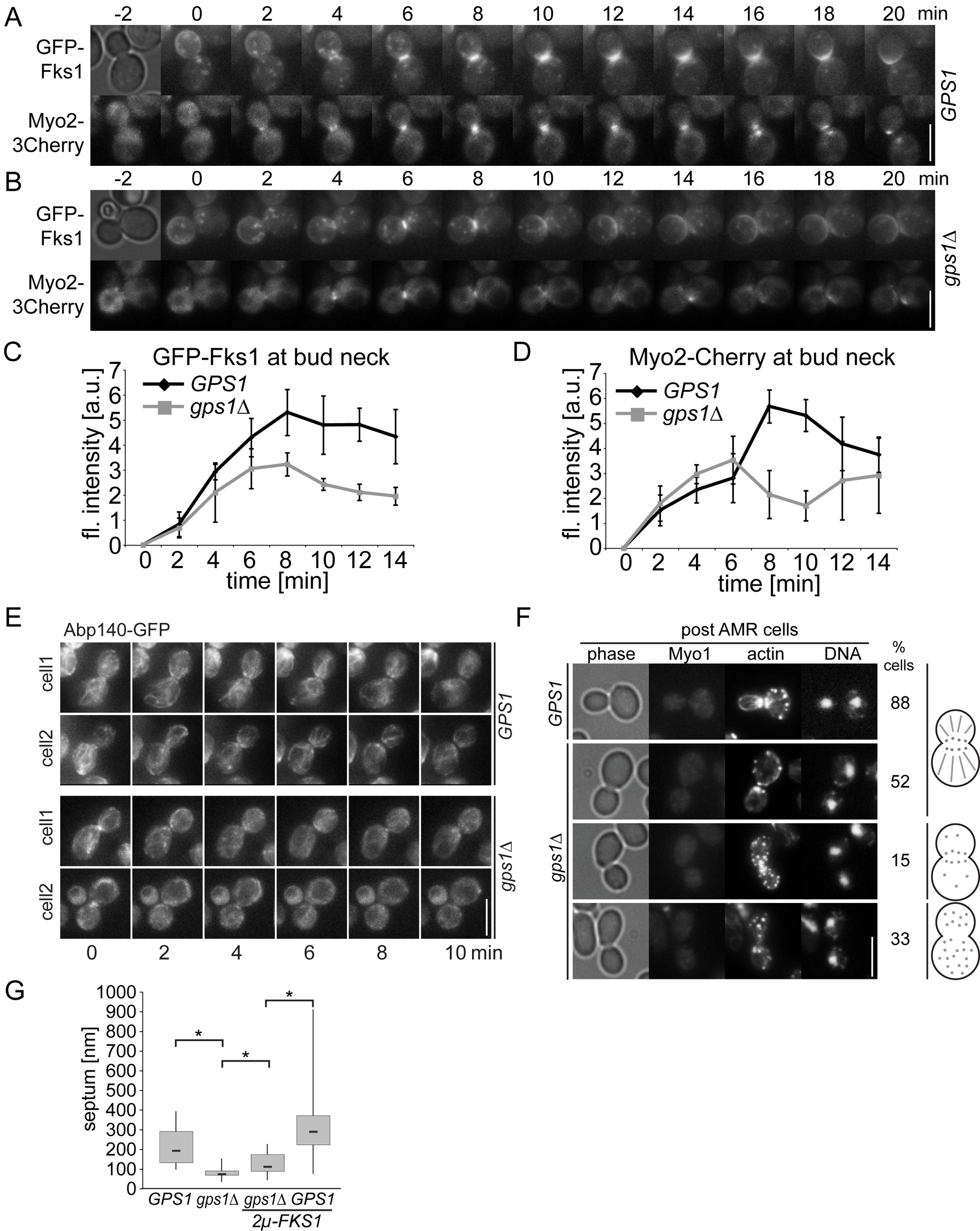

Supplement: Figure S5 — Gps1 is required to maintain Rho1-dependent polarization at the bud neck after actomyosin ring contraction. (A and B) Time-lapse series show the co-localization of GFP-Fks1 and Myo2-3Cherry at the bud neck during cytokinesis in wild-type (A) and gps1Δ (B) cells. (C and D) Quantification of GFP-Fks1 and Myo2-3Cherry at the bud neck in wild-type (n = 3) and gps1Δ (n = 3) cells. Error bars show the standard deviation. (E) The behavior of actin cables (labeled with Abp140-GFP) was analyzed by time-lapse microscopy in wild-type and gps1Δ cells. At the first time point, Abp140-GFP localizes at the bud neck as a ring (white arrowhead) (defined as t = 0). At later time points, actin cables toward the bud neck are visible in wild-type but not in gps1Δ cells. (F) Actin patches and cables were labeled with rhodamine-phalloidine and inspected in large budded MYO1-GFP cells (in the presence or absence of GPS1) that underwent AMR contraction (absence of Myo1-GFP at the bud neck). The percentage of cells with the indicated pattern of actin cables is indicated (n = 150–200 cells per strain). (G) The thickness of the septum (primary septum+secondary septum) of GPS1 and gps1Δ without or with overexpression of FKS1 (endogenous promoter from a 2 µ-based plasmid, 2 µ-FKS1) was quantified by electron microscopy analysis (GPS1, n = 10; gps1Δ, n = 14; gps1Δ 2 µ-FKS1, n = 14; GPS1 2 µ-FKS1, n = 35). PS, primary septum; SS, secondary septum. Scale bars: 5 µm. (TIF) [file pbio.1001495.s005.tif]

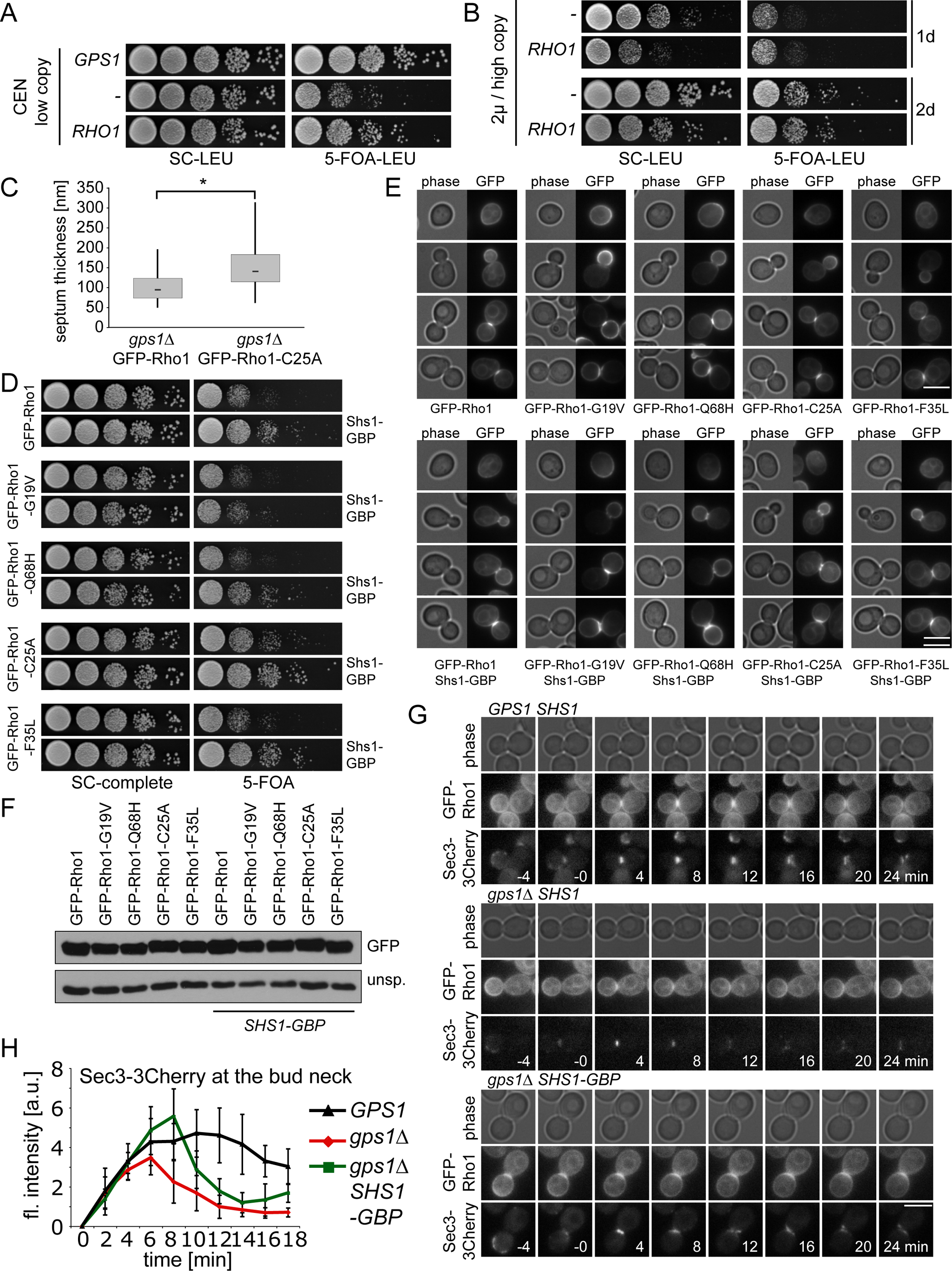

Supplement: Figure S6 — Rho1 rescue experiments of gps1Δ cells. (A) Serial dilutions of the gps1Δ URA3-GPS1 cells empty or carrying GPS1- or RHO1-containing low-copy plasmids (CEN, LEU2). 5-FOA selects against the URA3-containing plasmid. (B) Serial dilutions of the gps1Δ URA3-GPS1 cells carrying an empty or a RHO1 high-copy plasmid (2 µ, LEU2). (C) The thickness of the septum (primary septum+secondary septum) of gps1Δ GFP-RHO1 (n = 25) and gps1Δ GFP-rho1-C25A (n = 27) was quantified by electron microscopy analysis. “*” indicates p<0.01. Scale bars: 5 µm. (D) Serial dilutions of gps1Δ cells carrying GFP-RHO1, GFP-rho1-G19V, GFP-rho1-Q68H, GFP-rho1-C25A, and GFP-rho1-F35L with and without SHS1-GBP. (E) Still images show the localization of GFP-Rho1, GFP-Rho1-G19V, GFP-Rho1-Q68H, GFP-Rho1-C25A, and GFP-Rho1-F35L in cells with or without SHS1-GBP at different cell cycle stages. (F) Immunoblot shows protein levels of GFP-Rho1, GFP-Rho1-G19V, GFP-Rho1-Q68H, GFP-Rho1-C25A, and GFP-Rho1-F35L with and without SHS1-GBP. An unspecific signal of the GFP antibody was used as a loading control. (G) Localization of Sec3-3Cherry and GFP-Rho1 at the bud neck in wild-type, gps1Δ, and gps1Δ SHS1-GBP cells. (H) Quantification of Sec3-3Cherry in wild-type (n = 5), gps1Δ (n = 5), and gps1Δ SHS1-GBP (n = 5) cells expressing GFP-RHO1. Error bars show the standard deviation. (TIF) [file pbio.1001495.s006.tif]

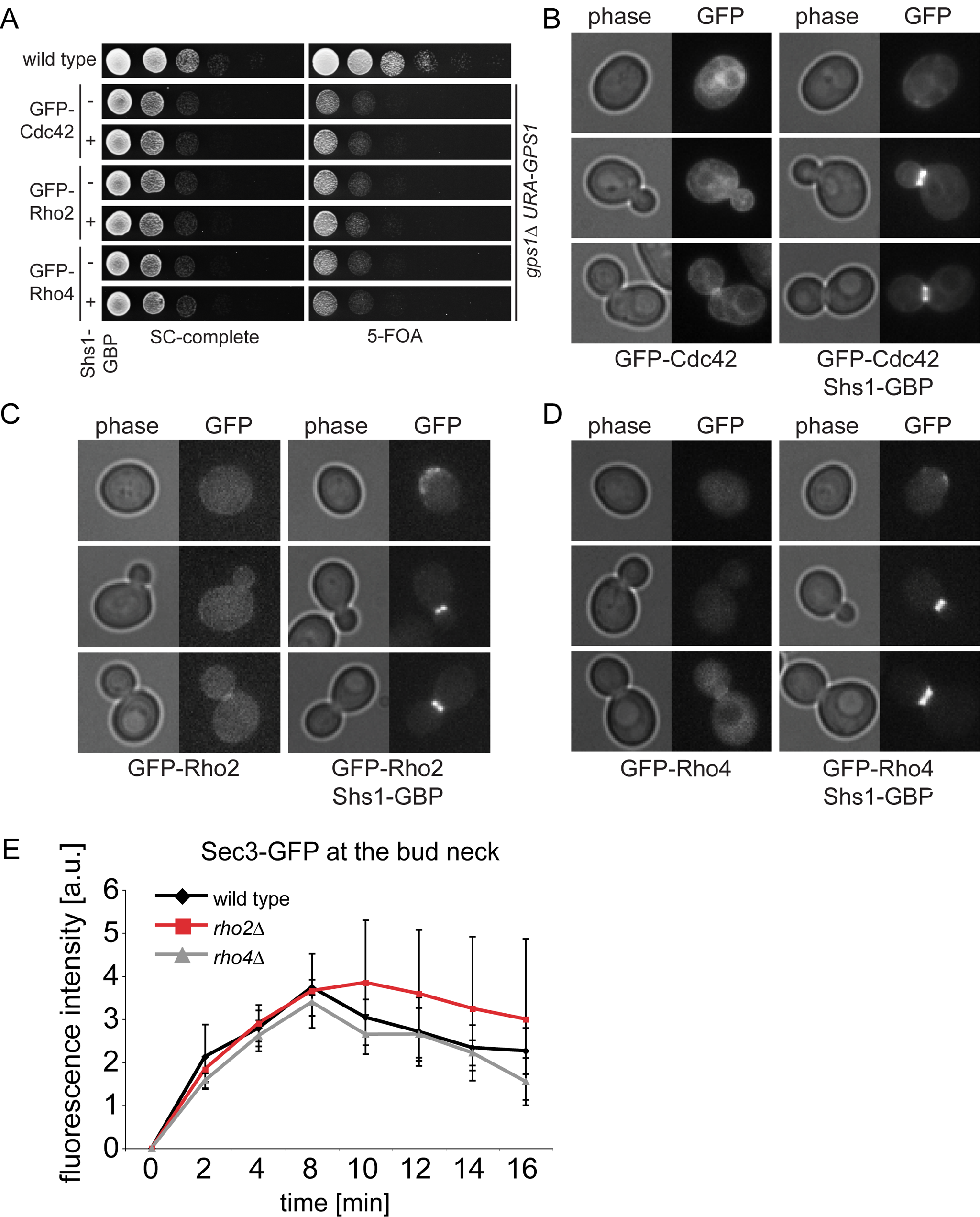

Supplement: Figure S7 — Rho2, Rho4, and Cdc42 are not involved in the Gps1-mediated septum formation pathway. (A) Serial dilutions of gps1Δ cells carrying GFP-CDC42, GFP-RHO2, or GFP-RHO4 with and without SHS1-GBP. (B–D) Still images of gps1Δ cells carrying GFP-CDC42, GFP-RHO2, or GFP-RHO4 with and without SHS1-GBP. (E) Quantification of time-lapse analysis of Sec3-GFP recruitment to the cell division site in wild-type, rho2Δ, and rho4Δ cells. Error bars show the standard deviation. Scale bar: 5 µm. (TIF) [file pbio.1001495.s007.tif]

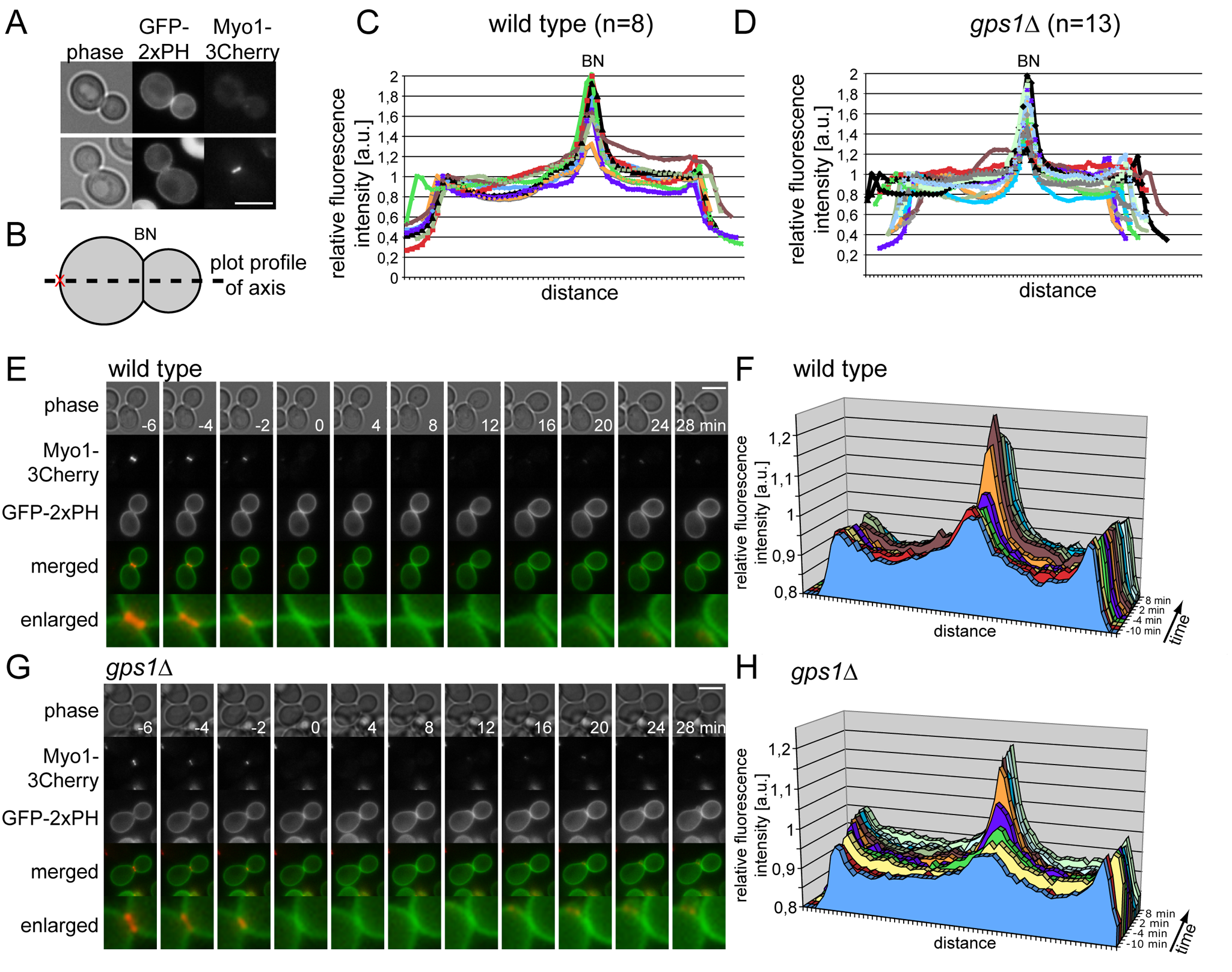

Supplement: Figure S8 — Gps1 is not required for PIP2 accumulation at the bud neck. (A) PIP2 (stained with GFP-2xPH) concentrates at the bud neck after (top panel) but not before (lower panel) AMR (Myo1) contraction. (B–D) Quantification of PIP2 at the bud neck of wild-type and gps1Δ cells. Scheme representation (B) and plot profiles (C and D) of PIP2 (GFP-2xPH fluorescence intensity) at the bud neck in wild-type and gps1Δ cells. BN, bud neck region. (E–H) Time-lapse series showing PIP2 (GFP-2xPH) localization during cytokinesis in wild-type (E and F) and gps1Δ (G and H) cells. (F) and (H) show the quantification of (E) and (G), respectively. Scale bars: 5 µm. (TIF) [file pbio.1001495.s008.tif]

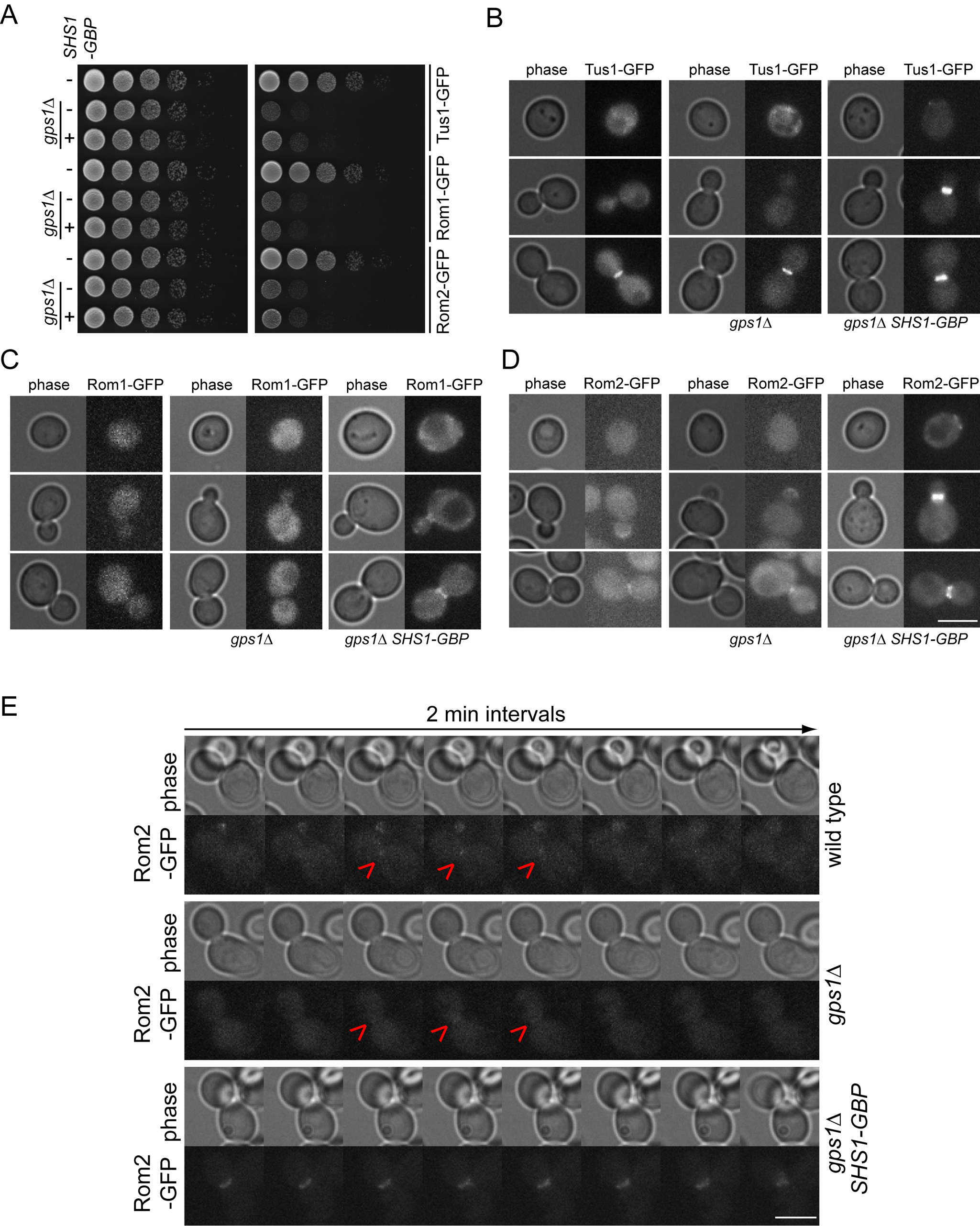

Supplement: Figure S9 — Gps1 is not required for Rho1 GEF localization at the bud neck. (A) Growth of serial dilutions of gps1Δ and gps1Δ SHS1-GBP cells carrying the Rho1 GEFs Tus1-GFP, Rom1-GFP, or Rom2-GFP. Note that Shs1-GBP tethers the GFP fusion protein to the bud neck constitutively. (B–D) Still images for the Rho1 GEFs Tus1-GFP, Rom1-GFP, or Rom2-GFP in wild-type, gps1Δ, and gps1Δ SHS1-GBP cells in different cell cycle stages. (E) Time-lapse series are shown for the localization of Rom2-GFP at the bud neck in wild-type, gps1Δ, and gps1Δ SHS1-GBP cells. Note that Rom2-GFP gives a dim signal at the bud neck in time-lapse analysis (red arrowhead) in both wild-type and gps1Δ cells. Scale bars: 5 µm. (TIF) [file pbio.1001495.s009.tif]

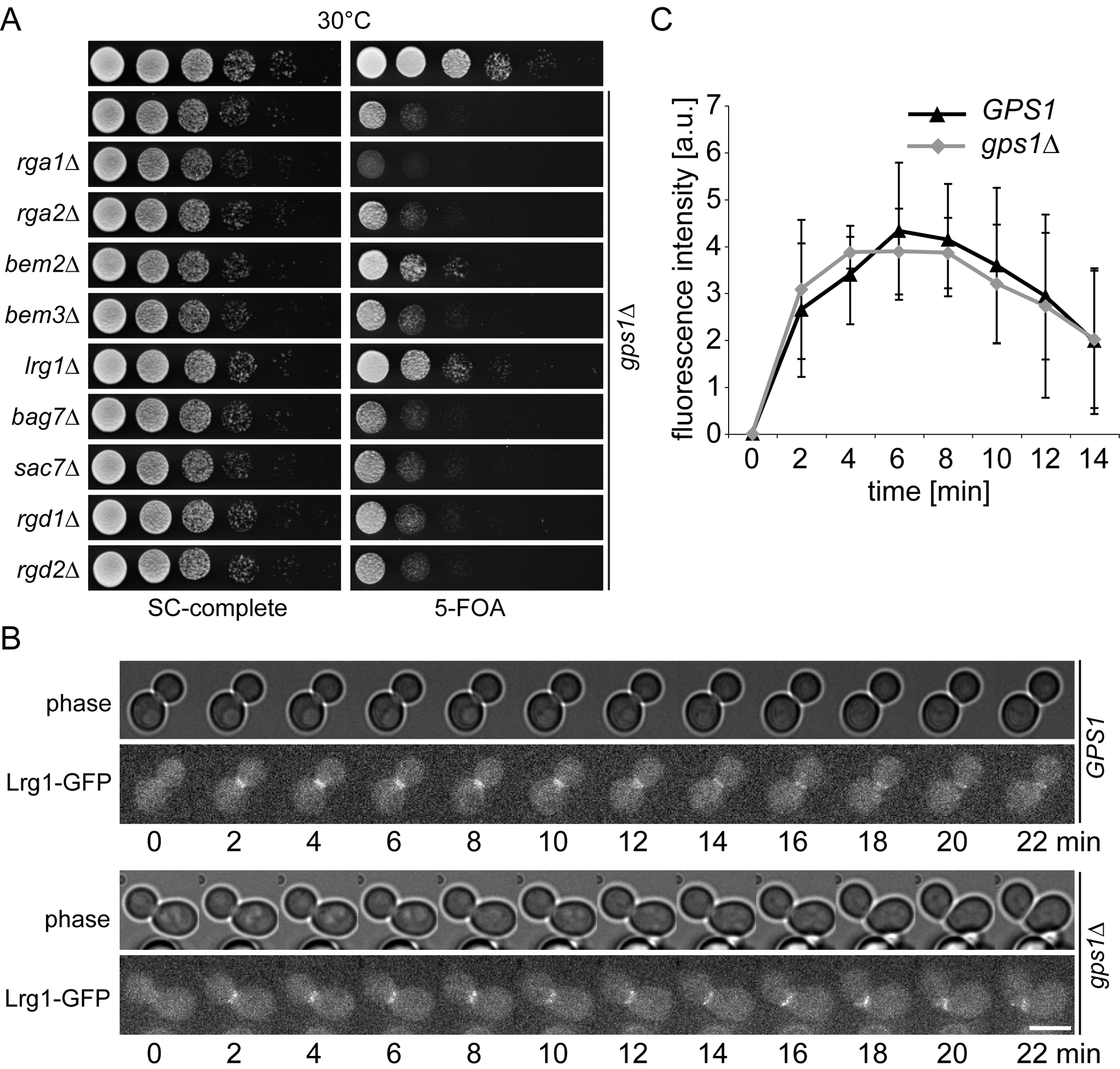

Supplement: Figure S10 — Rho GTPase GAPs and their involvement in the Gps1 pathway. (A) Growth of serial dilutions of gps1Δ cells with and without additional deletion of Rho GTPase GAPs as indicated. (B) Time-lapse series show Lrg1-GFP localization at the cell division site in wild-type and gps1Δ cells. (C) Graph shows the quantification of Lrg1-GFP signals at the bud neck in wild-type (n = 4) and gps1Δ (n = 3) cells. Error bars represent the standard deviation. (TIF) [file pbio.1001495.s010.tif]

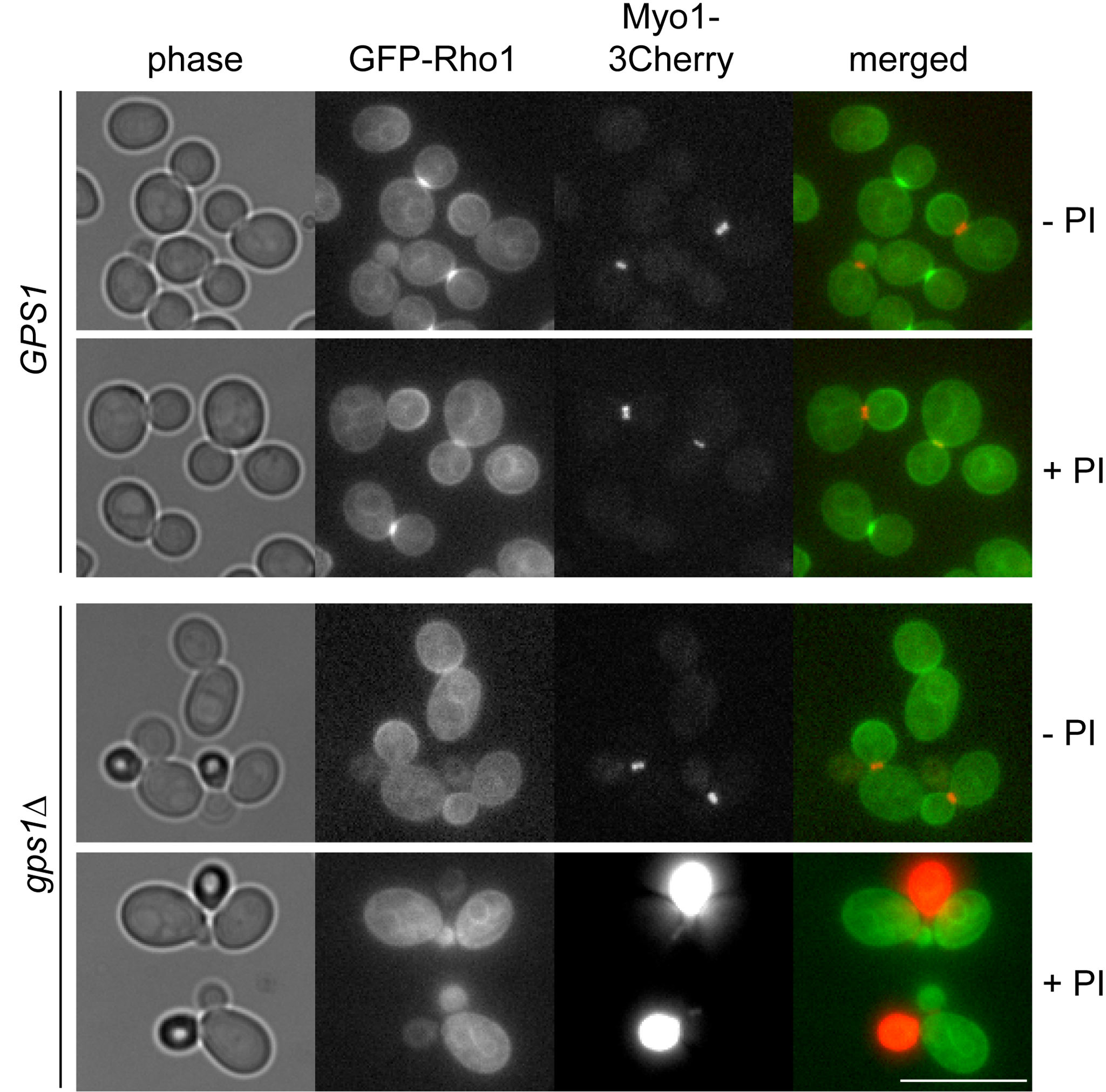

Supplement: Figure S11 — Cell death analysis of gps1Δ cells. Wild-type and gps1Δ cells with MYO1-3Cherry and GFP-RHO1 are stained with the cell death marker propidium iodide (PI). Scale bar: 10 µm. (TIF) [file pbio.1001495.s011.tif]

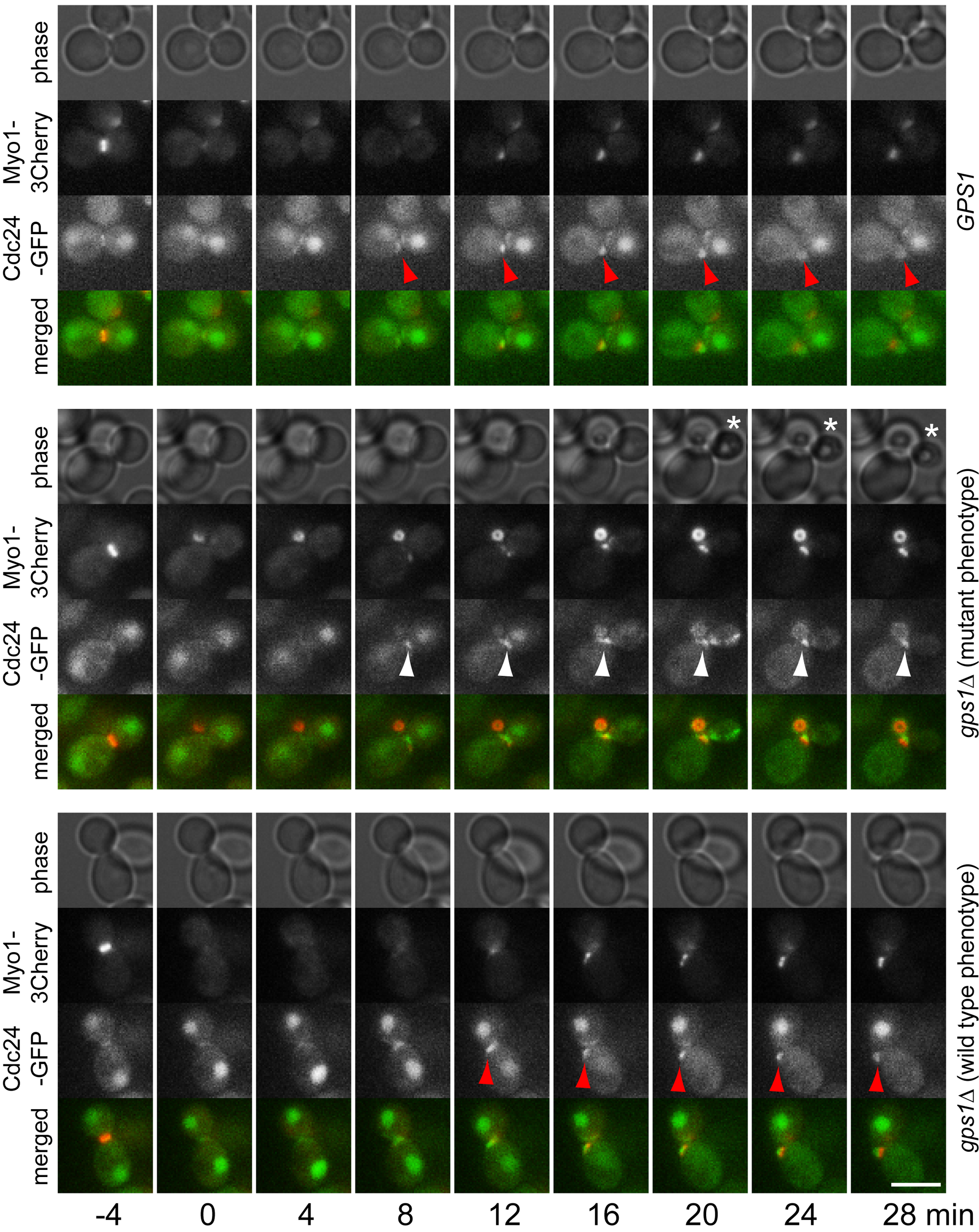

Supplement: Figure S12 — Cdc24 localization in gps1Δ cells. Time-lapse series show the localization of Cdc24-GFP in comparison to Myo1-3Cherry in wild-type and gps1Δ cells. Cdc24 accumulation at the bud neck (white arrowheads) and adjacent to the bud neck (red arrowheads) is depicted. White asterisks mark a dead daughter cell. (TIF) [file pbio.1001495.s012.tif]

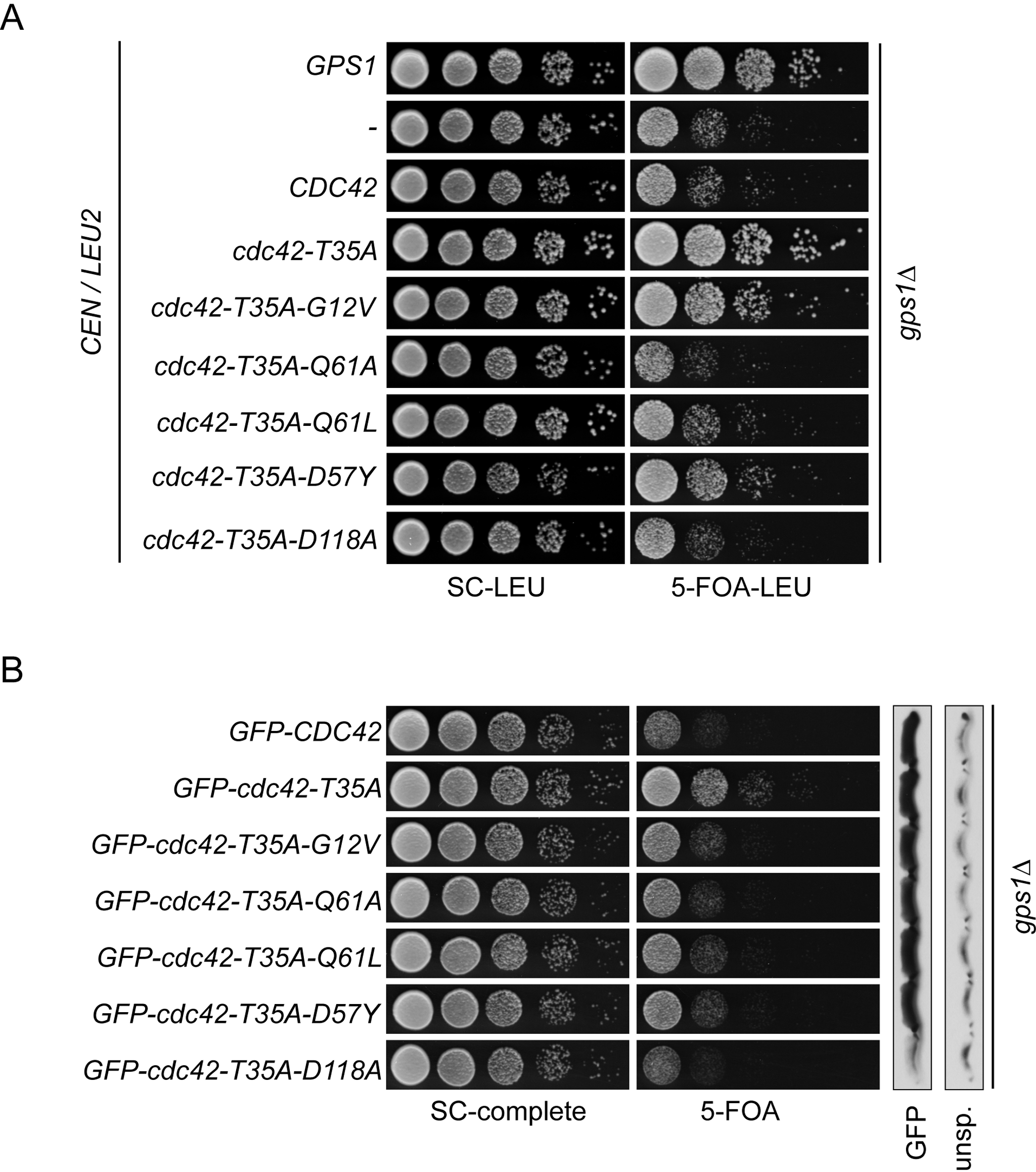

Supplement: Figure S13 — Cdc42-T35A rescue of gps1Δ is not consistently supported by locking Cdc42 in the GTP- or GDP-bound form. (A and B) Serial dilutions of gps1Δ cells expressing different CDC42 mutants from a low-copy plasmid (A) or stably integrated into the genome (B). Protein levels are shown. With the exception of GFP-Cdc42-T35A-D118A, the protein amounts of all mutants are comparable to wild-type GFP-Cdc42. Differences between (A) and (B) may result from different expression levels. (TIF) [file pbio.1001495.s013.tif]

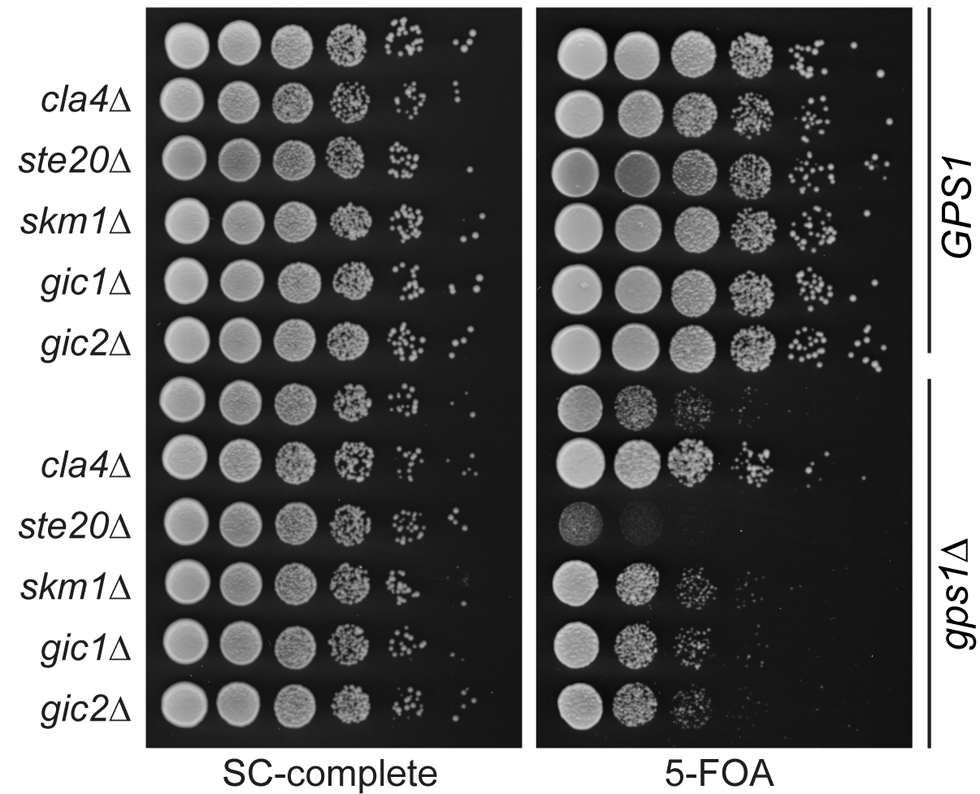

Supplement: Figure S14 — Genetic interaction between GPS1 and Cdc42 effectors. Growth of wild-type and gps1Δ cells upon deletion of the indicated Cdc42 effectors. (TIF) [file pbio.1001495.s014.tif]

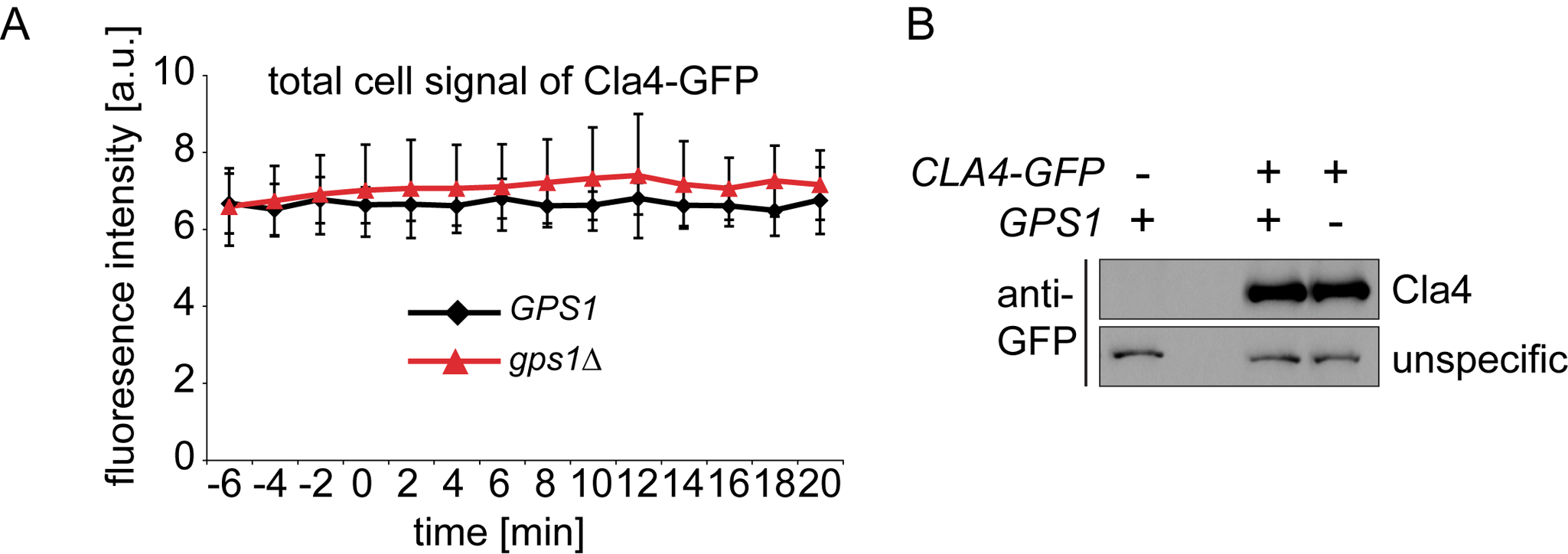

Supplement: Figure S15 — Cla4 localization in gps1Δ cells. (A) Quantification of the total cell signal of Cla4-GFP in a time-lapse series in GPS1 (n = 4) and gps1Δ (n = 4) cells. Error bars show the standard deviation. (B) Protein amounts of Cla4-GFP in GPS1 and gps1Δ cells determined by immunoblot. An unspecific signal was used as a loading control. (TIF) [file pbio.1001495.s015.tif]
