# Supplementary material for: A Safeguard Mechanism Regulates Rho GTPases to Coordinate Cytokinesis with the Establishment of Cell Polarity
Source: PLoS Biol. 2013 Feb 26;11(2):e1001495. doi: 10.1371/journal.pbio.1001495 (PMC3582507; doi:10.1371/journal.pbio.1001495)
Supplement: Table S1 — Rescue of gps1Δ defects. (DOC) [file pbio.1001495.s016.doc]

**Table S1. Rescue of *gps1∆*** defects.

| **Genotype** | **Genetic manipulation** | **Effect** | **Growth** | **SS thickness (median in nm)** | **Budding inside the old bud neck** | **Pathway** |
| --- | --- | --- | --- | --- | --- | --- |
| ***GPS1*** | ***-*** |  | **+++** | **193-245 (+)** | **0 %** |  |
| ***GPS1*** | *lrg1∆* | deletion of Rho1 GAP, increased Rho1 activity | +++ | 413 | 0 % | Rho1 |
| ***GPS1*** | *SHS1-GBP*  *GFP-RHO1* | tethering of Rho1 to the bud neck | +++ | 376 | 0 % | Rho1 |
| ***GPS1*** | pRS426*-FKS1* | increased Fks1 levels | n.d. | 289 | n.d. | Rho1 |
| ***GPS1*** | *cla4∆* | deletion | ++ | 256 | 0 % | Cdc42 |
| ***GPS1*** | *bud1∆* | bipolar budding | +++ | 278 | 0 % |  |
| ***gps1∆*** | ***-*** |  | **+** | **74-105 (+)** | **30-40 % (+)** |  |
| ***gps1∆*** | pRS315*-RHO1* | increased Rho1 levels | ++ | n.d. | n.d. | Rho1 |
| ***gps1∆*** | *GFP-rho1-C25A* | increased Rho1 activity | ++ | 141* | n.d. | Rho1 |
| ***gps1∆*** | *SHS1-GBP GFP-RHO1* | tethering of Rho1 to the bud neck | ++ | 136* | 46 % | Rho1 |
| ***gps1∆*** | *lrg1∆* | deletion of Rho1 GAP, increased Rho1 activity | ++ | 270* | 38 % | Rho1 |
| ***gps1∆*** | pRS426*-FKS1* | increased Fks1 levels | n.d. | 112* | n.d. | Rho1 |
| ***gps1∆*** | *cdc42-T35A* | impaired interaction with effectors | ++ | 104 | 0 % | Cdc42 |
| ***gps1∆*** | *cla4∆* | deletion | ++ | 151 | 0 % | Cdc42 |
| ***gps1∆*** | *cla4-kd* | kinase dead | ++ | n.d. | n.d. | Cdc42 |
| ***gps1∆*** | *cla4-crib-m1* | impaired interaction with Cdc42 | ++ | n.d. | n.d. | Cdc42 |
| ***gps1∆*** | *cla4-crib-m2* | impaired interaction with Cdc42 | ++ | n.d. | n.d. | Cdc42 |
| ***gps1∆*** | *ste20∆* | deletion | - | n.d. | n.d. | Cdc42 |
| ***gps1∆*** | *ste20-kd* | kinase dead | - | n.d. | n.d. | Cdc42 |
| ***gps1∆*** | *ste20-crib-m1* | impaired interaction with Cdc42 | - | n.d. | n.d. | Cdc42 |
| ***gps1∆*** | *ste20-crib-m2* | impaired interaction with Cdc42 | - | n.d. | n.d. | Cdc42 |
| ***gps1∆*** | *bud1∆* | bipolar budding | ++ | 89 nm | 0 % |  |

SS, secondary septum; n.d., not determined; (+), values from different experiments; *, significant difference to *gps1∆* control.
