# Supplementary material for: A Safeguard Mechanism Regulates Rho GTPases to Coordinate Cytokinesis with the Establishment of Cell Polarity
Source: PLoS Biol. 2013 Feb 26;11(2):e1001495. doi: 10.1371/journal.pbio.1001495 (PMC3582507; doi:10.1371/journal.pbio.1001495)
Supplement: Table S2 — Yeast strains used in this study. (DOC) [file pbio.1001495.s017.doc]

**Table S2. Yeast strains used in this study**

name genotype source

APY218 ESM356-1 *MYO1-3CHERRY-HIS3MX6* this study

APY226 ESM356-1 *GPS1-2GFP-kanMX6 SPC42-eqFP611-HIS3MX6*

*MYO1-3CHERRY-hphNT1*  this study

BY4741 *MATa his3Δ1 leu2Δ0 met15Δ0 ura3Δ0* this study

ESM356-1 *MATa* *ura3-52 leu2∆1 his3∆200 trp1∆63* [1]

FY1679 *MATa/alpha ura3-52/ura3-52 trp1∆63/TRP1 leu2∆1/LEU2 his3∆200/HIS3*  [2]

MFY323 YPH499 *∆gps1::HIS3MX6* this study

MFY330 YPH499 *∆hof1::TRP1* pRS316*-HOF1* this study

MFY337 YPH499 *∆hof1::TRP1* pRS316*-HOF1 ∆gps1::HIS3MX6* this study

MFY1553 ESM356-1 *URA3-GFP-FKS1::FKS1 MYO2-3CHERRY-HIS3MX6* this study

MFY1554 ESM356-1 *URA3-GFP-FKS1::FKS1 ∆gps1::hphNT1 MYO2-3CHERRY-HIS3MX6* this study

MFY1557 ESM356-1 *LRG1-GFP-klTRP1* this study

MFY1558 ESM356-1 *LRG1-GFP-klTRP1 ∆gps1::hphNT1* this study

MFY1563 ESM356-1 *ABP140-GFP-klTRP1*  this study

MFY1564 ESM356-1 *ABP140-GFP-klTRP1 ∆gps1::hphNT1* this study

MFY1565 ESM356-1 *CHS2-GFP-klTRP1* this study

MFY1566 ESM356-1 *CHS2-GFP-klTRP1 ∆gps1::hphNT1* this study

MFY1567 ESM356-1 *BNI1-GFP-klTRP1* this study

MFY1568 ESM356-1 *BNI1-GFP-klTRP1 ∆gps1::hphNT1* this study

MFY1570 ESM356-1 *SEC3-GFP-klTRP1* this study

MFY1571 ESM356-1 *SEC3-GFP-klTRP1 ∆gps1::hphNT1* this study

MFY1586 ESM356-1 *SEC3-GFP-klTRP1 ∆rho2::HIS3MX6* this study

MFY1587 ESM356-1 *SEC3-GFP-klTRP1 ∆rho4::HIS3MX6* this study

MFY1588 ESM356-1 *∆gps1::hphNT1* pRS316-*GPS1* this study

MFY1595 ESM356-1 *MYO1-3CHERRY-HIS3MX6* *ura3-52::*pRS306*-TUB1-GFP* this study

MFY1596 ESM356-1 *MYO1-3CHERRY-HIS3MX6 ∆gps1::hphNT1*

*ura3-52::*pRS306*-TUB1-GFP* this study

MFY1597 ESM356-1 *MYO1-3CHERRY-HIS3MX6 leu2∆1::*pRS305*-GFP-RHO1* this study

MFY1613 *MATa his3∆1 leu2∆0 met15∆0 ura3∆0* *GPS1-TAP-HIS3MX6*

*leu2∆1::*pRS305*-3HA-CDC42* this study

MFY1633 ESM356-1 *∆gps1::hphNT1* pRS316*-GPS1 leu2∆1::*pRS305*-GFP-RHO1* this study

MFY1634 ESM356-1 *∆gps1::hphNT1* pRS316*-GPS1 leu2∆1::*pRS305*-GFP-rho1-Q68H* this study

MFY1643 ESM356-1 *∆gps1::hphNT1* pRS316*-GPS1 leu2∆1::*pRS305*-GFP-RHO1*

*SHS1-GBP-kanMX6* this study

MFY1644 ESM356-1 *∆gps1::hphNT1* pRS316*-GPS1 leu2∆1::*pRS305*-GFP-rho1-Q68H*

*SHS1-GBP-kanMX6* this study

MFY1647 ESM356-1 *∆rga1::HIS3MX6* this study

MFY1651 ESM356-1 *∆lrg1::HIS3MX6* this study

MFY1656 ESM356-1 *∆gps1::hphNT1* pRS316*-GPS1 ∆rga1::HIS3MX6* this study

MFY1657 ESM356-1 *∆gps1::hphNT1* pRS316*-GPS1 ∆rga2::HIS3MX6* this study

MFY1658 ESM356-1 *∆gps1::hphNT1* pRS316*-GPS1 ∆bem2::HIS3MX6* this study

MFY1659 ESM356-1 *∆gps1::hphNT1* pRS316*-GPS1 ∆bem3::HIS3MX6* this study

MFY1660 ESM356-1 *∆gps1::hphNT1* pRS316*-GPS1 ∆lrg1::HIS3MX6* this study

MFY1661 ESM356-1 *∆gps1::hphNT1* pRS316*-GPS1 ∆bag7::HIS3MX6* this study

MFY1662 ESM356-1 *∆gps1::hphNT1* pRS316*-GPS1 ∆sac7::HIS3MX6* this study

MFY1663 ESM356-1 *∆gps1::hphNT1* pRS316*-GPS1 ∆rgd1::HIS3MX6* this study

MFY1664 ESM356-1 *∆gps1::hphNT1* pRS316*-GPS1* *∆rgd2::HIS3MX6* this study

MFY1666 ESM356-1 *MYO1-GFP-klTRP1 URA3::*YIp211*-GIC2-PBD-RFP* this study

MFY1668 ESM356-1 *MYO1-3CHERRY-hphNT1 leu2∆1::*pRS305*-GFP-CDC42*

*∆gps1::HIS3MX6* this study

MFY1671 ESM356-1 *MYO1-3CHERRY-HIS3MX6 leu2∆1::*pRS305*-GFP-RHO1* *∆gps1::hphNT1* this study

MFY1674 ESM356-1 *leu2∆1::*pRS305*-GFP-CDC42 ∆gps1::hphNT1* pRS316*-GPS1* this study

MFY1675 ESM356-1 *leu2∆1::*pRS305*-GFP-RHO2 ∆gps1::hphNT1* pRS316*-GPS1* this study

MFY1677 ESM356-1 *leu2∆1::*pRS305*-GFP-RHO4 ∆gps1::hphNT1* pRS316*-GPS1* this study

MFY1680 ESM356-1 *MYO1-GFP-klTRP1 URA3::*YIp211*-GIC2-PBD-RFP* *∆gps1::hphNT1* this study

MFY1682 ESM356-1 *∆gps1::hphNT1* pRS316*-GPS1 ∆cla4::HIS3MX6* this study

MFY1683 ESM356-1 *leu2∆1::*pRS305*-GFP-CDC42 ∆gps1::hphNT1* pRS316*-GPS1*

*SHS1-GBP-kanMX6* this study

MFY1684 ESM356-1 *leu2∆1::*pRS305*-GFP-RHO2 ∆gps1::hphNT1* pRS316*-GPS1*

*SHS1-GBP-kanMX6* this study

MFY1686 ESM356-1 *leu2∆1::*pRS305*-GFP-RHO4 ∆gps1::hphNT1* pRS316*-GPS1*

*SHS1-GBP-kanMX6*  this study

MFY1688 ESM356-1 *∆cla4::HIS3MX6* this study

MFY1694 ESM356-1 *∆ste20::HIS3MX6* this study

MFY1695 ESM356-1 *∆skm1::HIS3MX6* this study

MFY1696 ESM356-1 *∆gic1::HIS3MX6* this study

MFY1697 ESM356-1 *∆gic2::HIS3MX6* this study

MFY1698 ESM356-1 *∆gps1::hphNT1* pRS316*-GPS1 ∆ste20::HIS3MX6* this study

MFY1699 ESM356-1 *∆gps1::hphNT1* pRS316*-GPS1 ∆skm1::HIS3MX6* this study

MFY1700 ESM356-1 *∆gps1::hphNT1* pRS316*-GPS1 ∆gic1::HIS3MX6* this study

MFY1701 ESM356-1 *∆gps1::hphNT1* pRS316*-GPS1 ∆gic2::HIS3MX6* this study

MFY1711 ESM356-1 *∆gps1::hphNT1* pRS316*-GPS1 ∆axl1::HIS3MX6* this study

MFY1712 ESM356-1 *∆gps1::hphNT1* pRS316*-GPS1 ∆axl2::HIS3MX6* this study

MFY1713 ESM356-1 *∆gps1::hphNT1* pRS316*-GPS1 ∆bud1::HIS3MX6*  this study

MFY1714 ESM356-1 *∆gps1::hphNT1* pRS316*-GPS1 ∆bud2::HIS3MX6* this study

MFY1715 ESM356-1 *∆gps1::hphNT1* pRS316*-GPS1 ∆bud3::HIS3MX6* this study

MFY1716 ESM356-1 *∆gps1::hphNT1* pRS316*-GPS1 ∆bud4::HIS3MX6* this study

MFY1717 ESM356-1 *∆gps1::hphNT1* pRS316*-GPS1 ∆bud5::HIS3MX6* this study

MFY1718 ESM356-1 *∆gps1::hphNT1* pRS316*-GPS1 ∆bud7::HIS3MX6* this study

MFY1727 ESM356-1 *leu2∆1::*pRS305*-GFP-cdc42-T35A ∆gps1::hphNT1* pRS316*-GPS1* this study

MFY1741 ESM356-1 *∆gps1::hphNT1* pRS316*-GPS1 leu2∆1::*pRS305*-GFP-CDC42*

*MYO1-3CHERRY-kanMX6*  this study

MFY1742 ESM356-1 *∆gps1::hphNT1* pRS316*-GPS1 leu2∆1::*pRS305*-GFP-cdc42-T35A*

*MYO1-3CHERRY-kanMX6* this study

MFY1749 ESM356-1 *leu2∆1::*pRS305*-GFP-RHO1 SEC3-3CHERRY-HIS3MX6*

MFY1753 ESM356-1 *leu2∆1::*pRS305*-GFP-RHO1 ∆gps1::hphNT1* pRS316*-GPS1*

*SEC3-3CHERRY-HIS3MX6*  this study

MFY1755 ESM356-*1 leu2∆1::*pRS305*-GFP-RHO1 ∆gps1::hphNT1* pRS316*-GPS1*

*SEC3-3CHERRY-HIS3MX6 SHS1-GBP-kanMX6* this study

MFY1764 ESM356-1 *∆gps1::hphNT1* pRS316*-GPS1 ∆ste20::HIS3MX6 ∆bud1::klTRP1* this study

MFY1765 ESM356-1 *∆gps1::hphNT1* pRS316*-GPS1 ∆ste20::HIS3MX6 ∆bud4::klTRP1* this study

MFY1766 ESM356-1 *MYO1-3CHERRY CLA4-GFP-klTRP1* this study

MFY1767 ESM356-1 *∆gps1::hphNT1 MYO1-3CHERRY CLA4-GFP-klTRP1* this study

MFY1780 *MATa/alpha ura3-52/ura3-52 leu2∆1/ leu2∆1 his3∆200/his3∆200 trp1∆63/TRP1*

*∆bud1::HIS3MX6/∆bud1::HIS3MX6 ∆gps1::natNT1/∆gps1::hphNT1*

pRS316*-GPS1* this study

MFY1781 *MATa/alpha ura3-52/ura3-52 leu2∆1/ leu2∆1 his3∆200/his3∆200 trp1∆63/TRP1*

*∆bud4::HIS3MX6/∆bud4::HIS3MX6 ∆gps1::natNT1/∆gps1::hphNT1*

pRS316*-GPS1*  this study

MFY1782 *MATa/alpha ura3-52/ura3-52 leu2∆1/ leu2∆1 his3∆200/his3∆200 trp1∆63/TRP1*

*∆bud7::HIS3MX6/∆bud7::HIS3MX6 ∆gps1::natNT1/∆gps1::hphNT1*

pRS316*-GPS1*  this study

MFY1783 *MATa/alpha ura3-52/ura3-52 leu2∆1/ leu2∆1 his3∆200/his3∆200 trp1∆63/TRP1*

*∆bud8::HIS3MX6/∆bud8::HIS3MX6 ∆gps1::natNT1/∆gps1::hphNT1*

pRS316*-GPS1* this study

MFY1785 *MATa/alpha ura3-52/ura3-52 leu2∆1/ leu2∆1 his3∆200/his3∆200 trp1∆63/TRP1*

*∆rax1::HIS3MX6/∆rax1::HIS3MX6 ∆gps1::natNT1/∆gps1::hphNT1*

pRS316*-GPS1*  this study

MFY1803 ESM356-1 *CDC24-3HA-klTRP1* this study

MFY1804 ESM356-1 *∆gps1::hphNT1* pRS316*-GPS1 CDC24-3HA-klTRP1* this study

MFY1805 ESM356-1 *∆gps1::hphNT1* pRS316*-GPS1 CDC24-3HA-klTRP1 ∆cla4::HIS3MX6* this study

MFY1824 ESM356-1 *MYO1-3CHERRY-kanMX6 CDC24-GFP-klTRP1* this study

MFY1825 ESM356-1 *MYO1-3CHERRY-kanMX6 CDC24-GFP-klTRP1 ∆gps1::hphNT1* this study

MFY1826 ESM356-1 *TUS1-GFP-klTRP1* this study

MFY1827 ESM356-1 *∆gps1::hphNT1* pRS316-*GPS1* *TUS1-GFP-klTRP1* this study

MFY1828 ESM356-1 *∆gps1::hphNT1* pRS316-*GPS1* *TUS1-GFP-klTRP1 SHS1-GBP-kanMX6* this study

MFY1829 ESM356-1 *ROM1-GFP-klTRP1* this study

MFY1830 ESM356-1 *∆gps1::hphNT1* pRS316-*GPS1* *ROM1-GFP-klTRP1* this study

MFY1831 ESM356-1 *∆gps1::hphNT1* pRS316-*GPS1* *ROM1-GFP-klTRP1 SHS1-GBP-kanMX6* this study

MFY1832 ESM356-1 *ROM2-GFP-klTRP1* this study

MFY1833 ESM356-1 *∆gps1::hphNT1* pRS316-*GPS1* *ROM2-GFP-klTRP1* this study

MFY1834 ESM356-1 *∆gps1::hphNT1* pRS316-*GPS1* *ROM2-GFP-klTRP1 SHS1-GBP-kanMX6* this study

MFY1840 ESM356-1 *GPS1-6HA-HIS3MX6 ∆swi5::hphNT1* this study

MFY2082 ESM356-1 *leu2∆1::*pRS305*-GFP-cdc42-T35A-D57Y ∆gps1::hphNT1* pRS316*-GPS1* this study

MFY2083 ESM356-1 *leu2∆1::*pRS305*-GFP-cdc42-T35A-Q61A ∆gps1::hphNT1* pRS316*-GPS1* this study

MFY2097 ESM356-1 *leu2∆1::*pRS305*-GFP-cdc42-T35A-Q61L ∆gps1::hphNT1* pRS316*-GPS1* this study

MFY2123 ESM356-1 *leu2∆1::*pRS305*-GFP-cdc42-T35A-G12V ∆gps1::hphNT1* pRS316*-GPS1* this study

MFY2124 ESM356-1 *leu2∆1::*pRS305*-GFP-cdc42-T35A-D118A ∆gps1::hphNT1* pRS316*-GPS1* this study

SGY37 *leu2 ADE2 ura3-52::URA3-lexA-op-LacZ his3 trp1* [3]

SHY1 ESM356-1 *SHS1-GFP-kanMX6* this study

SHY10 ESM356-1 *SHS1-GFP-kanMX6 ∆gps1::klTRP1* this study

SHY32 ESM356-1 *leu2∆1::*pRS305*-GFP-RHO1* this study

SHY40 ESM356-1 *∆gas1::klTRP1* this study

SHY46 ESM356-1 *GPS1-2GFP-kanMX6 SPC42-eqFP611-hphNT1* this study

SHY51 ESM356-1 *∆slt2::klTRP1* this study

SHY65 ESM356-1 *∆gps1::hphNT1* this study

SHY99 ESM356-1 *GPS1-2GFP-kanMX6 SPC42-eqFP611-hphNT1*

*SHS1-3CHERRY-HIS3MX6*  this study

SHY109 ESM356-1 *MYO1-3CHERRY-HIS3MX6 ∆gps1::hphNT1* this study

SHY117 ESM356-1 *MYO1-3CHERRY-hphNT1 leu2∆1::*pRS305*-GFP-CDC42* this study

W4275 BY4741 *∆slt2::natMX4* this study

W5144 BY4741 *GPS1-13MYC-natMX4 ∆pep4::URA3* this study

W5146 BY4741 *GPS1-13MYC-natMX4 ∆swi5::natMX4 ∆pep4::URA3* this study

W5853 BY4741 *∆swi5::kanMX4* this study

W5854 BY4741 *∆gps1::kanMX4* this study

W7918 BY4741 *∆swi5::kanMX4 ∆slt2::natMX4* this study

W7920 BY4741 *∆gps1::kanMX4 ∆slt2::natMX4* this study

W10771 *MATalpha ∆swi5::kanMX ∆can1::MFA1pr-HIS3-MFAlpha1pr-LEU2* this study

W10772 *MATalpha ∆swi5::kanMX ∆can1::MFA1pr-HIS3-MFAlpha1pr-LEU2*

pRS316-pTEF2*-MYC-GPS1* this study

YPH499 *MATa ura3-52 lys2-801amber ade2-101ochre trp1∆63 his3∆200 leu2∆1* [4]

YPH500 *MATalpha ura3-52 lys2-801amber ade2-101ochre trp1∆63 his3∆200 leu2∆1* [4]

**Literature**

1. Pereira G, Tanaka TU, Nasmyth K, Schiebel E (2001) Modes of spindle pole body inheritance and segregation of the Bfa1p-Bub2p checkpoint protein complex. Embo J 20: 6359-6370.

2. Winston F, Dollard C, Ricupero-Hovasse SL (1995) Construction of a set of convenient Saccharomyces cerevisiae strains that are isogenic to S288C. Yeast 11: 53-55.

3. Geissler S, Pereira G, Spang A, Knop M, Soues S, et al. (1996) The spindle pole body component Spc98p interacts with the gamma-tubulin-like Tub4p of Saccharomyces cerevisiae at the sites of microtubule attachment. Embo J 15: 3899-3911.

4. Sikorski RS, Hieter P (1989) A system of shuttle vectors and yeast host strains designed for efficient manipulation of DNA in Saccharomyces cerevisiae. Genetics 122: 19-27.
