# Supplementary material for: A Safeguard Mechanism Regulates Rho GTPases to Coordinate Cytokinesis with the Establishment of Cell Polarity
Source: PLoS Biol. 2013 Feb 26;11(2):e1001495. doi: 10.1371/journal.pbio.1001495 (PMC3582507; doi:10.1371/journal.pbio.1001495)
Supplement: Table S3 — Plasmids used in this study. (DOC) [file pbio.1001495.s018.doc]

**Table S3. Plasmids used in this study**

name genotype source

p297 pRS315-*CDC42* [1]

p487 pRS315-*cdc42-T35A*  [1]

pAP38 pRS316-*GPS1* this study

pCT21 pRS305-*3HA-CDC42* gift from T. Höfken

pGW069 YEp13-*STE20* gift from E. Schiebel

pILM21 pRS425-*RHO1* this study

pMB29 pRS305-*GFP-CDC42* gift from T. Höfken

pMB50 pRS305-*GFP-RHO1*  gift from T. Höfken

pMB51 pRS305-*GFP-RHO2* gift from T. Höfken

pMB53 pRS305-*GFP-RHO4* gift from T. Höfken

pMF567 pRS305-*GFP-rho1-G19V* this study

pMF568 pRS305-*GFP-rho1-Q68H* this study

pMF569 pRS305-*GFP-rho1-C25A* this study

pMF570 pRS305-*GFP-rho1-F35L* this study

pMF592 pRS305-*GFP-cdc42-T35A* this study

pMF603 pRS315-*cdc42-T35A-G12V* this study

pMF604 pRS315-*cdc42-T35A-D118A* this study

pMF605 pRS315-*GPS1* this study

pMF642 pRS315-*ste20-K649R* (ste20-kd) this study

pMF643 pRS315-*ste20-H345D-H348D* (ste20-crib-m2) this study

pMF644 pRS315-*ste20-337-340A* (ste20-crib-m1) this study

pMF633 pRS315-*CLA4-9MYC* this study

pMF645 pRS315-*cla4-K594R-9MYC* (cla4-kd) this study

pMF646 pRS315-*cla4-H192D-H194D-9MYC* (cla4-crib-m2) this study

pMF647 pRS315-*cla4-184-187A-9MYC* (cla4-crib-m1) this study

pMF705 pRS305-*GFP-cdc42-T35A-G12V* this study

pMF706 pRS305-*GFP-cdc42-T35A-D118A* this study

pMF766 pGEX-5X-1-*RHO1* this study

pMF767 pGEX-5X-1-*CDC42* this study

pMF772 pET28c-*gps1-aa1-290* this study

pMF936 pRS315-*cdc42-T35A-D57Y* this study

pMF937 pRS315-*cdc42-T35A-Q61A* this study

pMF940 pRS305-*GFP-cdc42-T35A-D57Y* this study

pMF941 pRS305-*GFP-cdc42-T35A-Q61A* this study

pMF942 pGEX-5X-1*-cdc42-D57Y* this study

pMF957 pRS315-*cdc42-T35A-Q61L* this study

pMF959 pRS305-*GFP-cdc42-T35A-Q61L* this study

pMF960 pGEX-5X-1*-cdc42-Q61L* this study

pMF962 pGEX-5X-1-*rho1-G19V* this study

pMF963 pGEX-5X-1-*rho1-G22A* this study

pMF973 pRS315-*RHO1* this study

pMF984 pRS425-*GFP-RHO1* this study

pMM5 pRS423-Gal1-*LexA-MYC* [2]

pMM6 pRS425-Gal1-*Gal4-HA* [2]

pRS305 integration, *LEU2* [3]

pRS306 integration, *URA3*  [3]

pRS315 *CEN*, *LEU2* [3]

pRS316 *CEN*, *URA3* [3]

pRS425 2µ, *LEU2* [4]

pRS426 2µ, *URA3* [4]

pTH35 pRS315-*STE20* [5]

pTH163 pRS425-*CLA4* gift from E. Schiebel

pWS1470 pRS316-pTEF2-*myc3-GPS1*-tCYC1 this study

pWS3161 pRS306-pFKS1-*GFP-FKS1-∆C-*tCYC1 this study

pWS-FKS1 pRS426-*FKS1* this study

YEP13 2µ, *LEU2* [6]

**Literature**

1. Ziman M, O'Brien JM, Ouellette LA, Church WR, Johnson DI (1991) Mutational analysis of CDC42Sc, a Saccharomyces cerevisiae gene that encodes a putative GTP-binding protein involved in the control of cell polarity. Mol Cell Biol 11: 3537-3544.

2. Geissler S, Pereira G, Spang A, Knop M, Soues S, et al. (1996) The spindle pole body component Spc98p interacts with the gamma-tubulin-like Tub4p of Saccharomyces cerevisiae at the sites of microtubule attachment. Embo J 15: 3899-3911.

3. Sikorski RS, Hieter P (1989) A system of shuttle vectors and yeast host strains designed for efficient manipulation of DNA in Saccharomyces cerevisiae. Genetics 122: 19-27.

4. Christianson TW, Sikorski RS, Dante M, Shero JH, Hieter P (1992) Multifunctional yeast high-copy-number shuttle vectors. Gene 110: 119-122.

5. Hofken T, Schiebel E (2002) A role for cell polarity proteins in mitotic exit. Embo J 21: 4851-4862.

6. Rose AB, Broach JR (1990) Propagation and expression of cloned genes in yeast: 2-microns circle-based vectors. Methods Enzymol 185: 234-279.
